# Sustaining Recovery After Low‐Intensity Treatment for Anxiety and Depression in NHS Talking Therapies: A Multiphase Participatory and Consensus‐Building Study of Stakeholder Priorities and Recommendations

**DOI:** 10.1155/da/9916526

**Published:** 2026-01-28

**Authors:** Saher Nawaz, Penny Bee, Cintia Faija

**Affiliations:** ^1^ School of Health Sciences, Division of Nursing, Midwifery and Social Work, Manchester Academic Health Science Centre, University of Manchester, Manchester, UK, manchester.ac.uk; ^2^ Department of Primary Care and Mental Health, Faculty of Health and Life Sciences, Institute of Population Health, University of Liverpool, Liverpool, UK, liv.ac.uk

## Abstract

Anxiety and depression affect over 500 million people globally. Despite the availability of effective low‐cost treatments, like those provided by NHS talking therapies (TT), over half of patients relapse within a year, highlighting the need to co‐develop solutions to maintain wellbeing and optimise healthcare. This study used a multiphase participatory design to synthesise evidence on relapse prevention and collaboratively develop evidence‐informed strategies for sustained mental health following low‐intensity treatment. The three‐phase project began by synthesising evidence from diverse sources. Phase 2 involved two patient and two NHS professional/key stakeholder co‐design workshops, using the RAND/UCLA appropriateness method. A sustained patient and public involvement (PPI) group reviewed and refined findings, co‐designing content for phase 3, which involved a mixed‐stakeholder online meeting to finalise key recommendations and priorities. Phase 1 identified 41 evidence‐based solutions for rating during phase 2. Across the four phase 2 workshops (*n* = 18), 24 solutions were rated as appropriate and necessary and ranked for priorities. These were refined with the PPI group into 13 core recommendations. These recommendations were incorporated into a relapse prevention model during the final workshop, forming a foundation to enhance post‐treatment support and inform clinical practice, service design, workforce training and policy. Preventing relapse and supporting wellbeing are essential for improving patient outcomes and reducing health inequalities. Identifying priorities across multiple levels lays the groundwork for a robust relapse prevention model that promotes sustained recovery. Future research should implement and evaluate the feasibility and impact of these recommendations in routine care.

## 1. Introduction

Anxiety and depression are among the most prevalent mental health conditions worldwide [1], affecting an estimated 280 million and 301 million individuals, respectively [2, 3]. In the United Kingdom, the NHS talking therapies (TT) for anxiety and depression (NHS TTad; formerly IAPT) programme was established in 2008 to increase access to evidence‐based psychological interventions and reduce the burden of common mental disorders [4]. NHS TTad follows a stepped‐care model, offering low‐intensity cognitive behavioural therapy (CBT)‐based interventions at Step 2 and high‐intensity therapies at Step 3 [5, 6].

While NHS TTad has demonstrated substantial clinical impact, with ~50% of patients achieving recovery at treatment completion [7], relapse remains a critical challenge. Longitudinal evidence from NHS TTad services shows that over half of those who recover relapse within a year—most within 6 months [8]. The WYLOW study reported relapse or recurrence in 65.8% of patients within 24 months following low‐intensity treatment [9], and ~14% of recovered individuals return for additional treatment within 5 years [10, 11], highlighting a ’revolving door’ phenomenon.

These findings underscore the need for strategies that not only treat symptoms but also maintain recovery and prevent relapse. While interventions such as mindfulness‐based cognitive therapy have demonstrated efficacy in preventing relapse following high‐intensity CBT [12, 13], there is limited evidence on relapse prevention following low‐intensity interventions [14].

Effective development of tools to support sustained recovery requires meaningful involvement of stakeholders (patients, clinicians and service designers) who bring vital experiential and contextual knowledge [15, 16]. Consensus methodologies can facilitate this process, enabling diverse stakeholder groups to interpret evidence collaboratively and shape practical solutions for implementation [17].

The use of structured intervention development frameworks, such as the Double Diamond Framework (Design [18]) and the Behaviour Change Wheel [19], provides a systematic basis for designing interventions that are both evidence‐informed and context‐sensitive [20, 21]. These frameworks have increasingly been combined to co‐produce scalable and sustainable tools for mental health services [22].

This study used a multiphase participatory design process to synthesise existing evidence and, in collaboration with stakeholders, co‐develop evidence‐based informed solutions to support sustained mental health following low‐intensity treatment. The aims of the study were as follows:

Phase 1: to synthesise existing evidence on factors contributing to mental health relapse following discharge from low‐intensity treatment, as well as the resources currently available to prevent relapse after guided self‐help.

Phase 2: to engage multiple stakeholder groups via a consensus‐building process to evaluate evidence‐based solutions and identify priorities for supporting sustained mental health over time (i.e., following clinical recovery).

Phase 3: to co‐produce a set of evidence‐based, stakeholder‐informed recommendations to enhance mental health support over time, with implications for future clinical practice, service design and policy development.

## 2. Methods

### 2.1. Co‐Improve Project Phases

#### 2.1.1. Phase 1: Evidence Synthesis

Evidence is crucial for developing evidence‐based solutions to a problem identified [23]. For the present study, this included a scoping review of the interventions and resources available to support recovery and prevent relapse following guided self‐help [14]. In addition, evidence included findings from two qualitative studies designed to understand routine care and current practices in NHS TTad targeting maintenance of recovery and relapse prevention within and after the end of treatment [24]. Evidence from multiple sources was synthesised, from which evidence‐based solutions were derived to be discussed and rated in the subsequent phase.

#### 2.1.2. Phase 2: Stakeholder Engagement via a Consensus‐Building Process

The stakeholder engagement phase aimed to (1) ensure development and improvements to routine care are informed by the experiences of those providing and utilising services [25], (2) agree on solutions that are most needed and appropriate to support long‐term recovery and wellbeing and (3) identify priorities for supporting sustained mental health over time.

Four co‐design collaborative online workshops were conducted, two with patients and two with professionals (i.e., practitioners, clinical service leads and policymakers).

The RAND/UCLA methodology was used to work in collaboration with different groups of stakeholders in the translation of evidence into practice to co‐develop an intervention. The RAND/UCLA provides a systematic approach to developing clinical practice guidelines [26] and is a reliable framework for healthcare decision‐making [27, 28], combining empirical evidence through expert opinions and facilitating participant discussions. The RAND/UCLA methodology assesses the appropriateness and necessity of evidence‐based elements derived from phase 1. The workshops were delivered online via Microsoft Teams, and data collection for the three‐round voting process of the RAND/UCLA was facilitated via Qualtrics [29].

#### 2.1.3. Phase 3: Shared Decision Making With Stakeholders

The shared decision‐making phase was aimed to (1) report on the findings from the co‐development workshops, highlighting similarities and differences across groups of stakeholders and between groups; (2) discuss priorities identified for inclusion in an intervention; (3) identify feasible and acceptable ways to develop and implement the multilevel intervention. An online workshop was conducted via Microsoft Teams with multiple stakeholders (i.e., patients, practitioners, academics and national leads). The workshop allowed for opportunities to discuss as a whole group but also in breakout rooms for each group of stakeholders (i.e., patients and professionals).

### 2.2. Eligibility Criteria and Recruitment

The eligibility criteria to participate in the qualitative studies of phase 1 and the workshops for phases 2 or 3 were as follows:

#### 2.2.1. Patients

Adults 18 years or over who had started low‐intensity treatment with case‐level depression (measured by a score of 10 or more on the patient health questionnaire‐9 [30] and/or anxiety (measured by a score of 8 or more on the generalised anxiety disorder‐7 (GAD‐7; [31]) and met the NHS TTad recovery criteria on the final session attended (i.e., scores below 10 and 8 on the PHQ‐9 and GAD‐7, respectively), and were discharged 6 months prior to research participation.

Patient recruitment was supported by NHS TTad services across five NHS Trusts within the North of England. The NHS staff at each site identified eligible patients from their records and approached patients via their preferred method of contact (telephone, email or post), sending them an invitation pack. Interested patients approached the research team directly or provided consent to NHS staff to share their details with the research team, who then approached them via telephone and/or email. Patients who had consented to be contacted for future research from phase 1 of the study were invited to participate in the following phases. Participants had an opportunity to ask questions, discuss their involvement and availability with the research team. Participants who agreed to participate provided informed consent online and completed an online demographics form before participation.

#### 2.2.2. Professionals and Other Key Stakeholders

Professionals were eligible to participate if they were trainees, a qualified or senior PWP who deliver low‐intensity interventions in NHS TTad services, an NHS TTad services lead or team manager, and NHS TTad national lead, a clinical academic delivering PWP training, or involved in NHS TTad research or influencing policy.

The recruitment of professionals and key stakeholders involved three channels: (1) via four of the five NHS Trusts that supported patient recruitment. The clinical lead disseminated the study details via their preferred method of communication (email or verbally during meetings), and, when invited, the research team advertised the project in multidisciplinary team meetings, (2) via social media, (3) eligible participants that consented to be contacted from other research studies were invited to participate by other researchers or by the research team.

All potential participants received an invitation pack with details about the study. Interested participants contacted the research team (via email or telephone). All participants provided informed consent online and completed an online demographics form before participating.

### 2.3. Data Collection

#### 2.3.1. Phase 1: Evidence Synthesis

Evidence from three sources was reviewed to develop evidence‐based solutions to support wellbeing and recovery following the end of low‐intensity treatment delivered in NHS TTad services. These included: (1) findings from a scoping review identifying three interventions and three tools on supporting recovery and preventing relapse following guided self‐help treatment for anxiety and/or depression [14], (2) findings from a qualitative study conducted with 23 eligible patients discharged from NHS TTad services, (3) a qualitative study exploring views of 25 professionals and key stakeholders on long‐term recovery within NHS TTad [24].

#### 2.3.2. Phase 2: Stakeholder Engagement via a Consensus‐Building Process

Interested participants were invited to an online workshop via MS Teams (professionals) or Zoom (patients). The workshop was scheduled for up to 5 h. The workshop allocated time for welcoming and introductions, project research background, discussion of findings from the evidence synthesis (phase 1), a group voting and ranking exercise, information about resources currently available to support relapse prevention, and brainstorming ideas for intervention format and implementation challenges. Workshops were facilitated by the principal investigator and the patient and public involvement (PPI) co‐applicant, supported by the research assistant. All workshops were recorded and transcribed, following participant consent.

During the workshop, participants completed a three‐round voting exercise informed by the RAND/UCLA method [28]. In round 1, participants rated the statements for appropriateness using a scale from 1 (not appropriate at all) to 9 (extremely appropriate). Statements were rated independently using an allocated ID. Before round 2, participants discussed and shared their thoughts about personal ratings related to statements in disagreement. Following the moderated discussion, participants re‐rated all the statements on appropriateness. In round 3, participants were asked to rate statements regarding necessity.

Following the 3 rounds of voting, participants ranked the agreed statements to identify priorities within the agreed solutions for supporting sustained mental health over time. Following the ranking exercise, participants discussed options for implementation of the solutions to assist its uptake and facilitation in routine practices in NHS TTad services.

Following the RAND/UCLA methodology, a sample size between 8 and 12 for each stakeholder group of experts was deemed appropriate to allow the panel to be large enough to account for diversity of representation while being small enough to facilitate everyone’s involvement in the discussions [28].

#### 2.3.3. Data Analysis

Following the RAND/UCLA methodologies guidelines, we adhered to the strict agreement criteria of A9S, defined as *‘all ratings fall within a single three-point region (1–3; 4–6; 7–9)*’ ([28], p.57). This criterion ensures robust consensus in the evaluation process, underscoring the rigour of our approach.

Data from the RAND/UCLA voting exercise were analysed descriptively using SPSS [32] to identify statements in agreement and disagreement among participants. Findings from round 1 were shared with participants visually using SPSS graphs. Findings from round 2 included all statements in which all participants showed agreement and were rated as appropriate. Those were then rated in round 3, and data was descriptively analysed to identify agreement across participants on necessity statements. Statements rated as necessary in round 3 were then ranked online (i.e., Mentimeter and Opinion X).

### 2.4. Smaller Sustained Group Work Between Phases With Members of the PPI Group

Following phase 2, three members of the PPI advisory group participated in smaller sustained group work comprised of two meetings. Group work focused on discussing the findings from phase 2 and informed the content and presentation of the findings for phase 3. The chief investigator and the PPI co‐applicant facilitated the group work, supported by the research assistant.

#### 2.4.1. Phase 3: Shared Decision‐Making With Stakeholders

##### 2.4.1.1. Procedure

The sample size was defined by a number considered large enough to account for diversity of representation and small enough to facilitate everyone’s involvement in the discussions. Recruitment was aimed at three–five patients and three–five professionals/other stakeholders. Interested participants were invited to participate in a 2‐h workshop which covered findings from phase 2, allowed for opportunities to discuss and brainstorm implementation challenges, and facilitators for a multilevel intervention (i.e., patients, professionals, training curriculum and policy) as a whole group but also in breakout rooms (i.e., patients and professionals). The workshop was recorded and transcribed following consent.

## 3. Results

**Figure 1:**
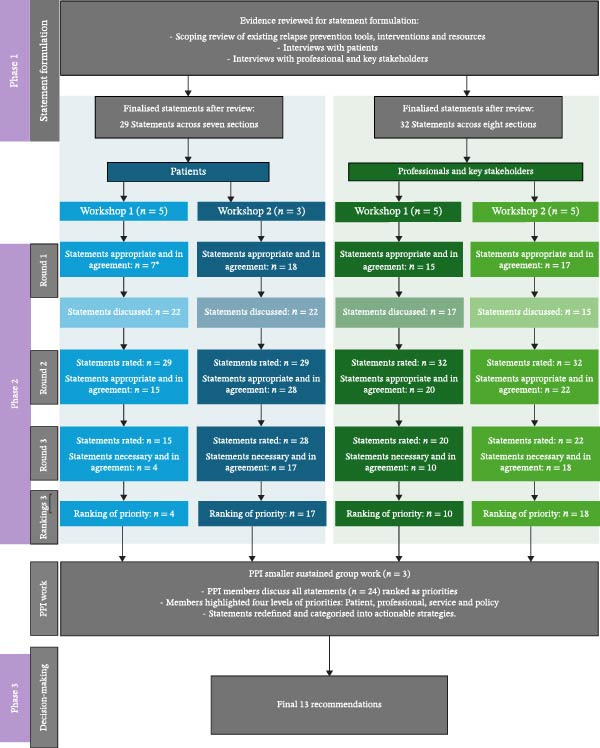
A flow chart of summary of results and statements ranked and rated in each round across the four workshops.

Figure 1 provides an overview of the findings across the three phases. The results are systematically organised by phase, demonstrating the iterative progression of the research and the development of emerging insights and recommendations throughout the study.

### 3.1. Phase 1: Evidence Synthesis

Evidence from multiple sources was extracted to inform statements on evidence‐based solutions to maintain wellbeing and prevent relapse following low‐intensity interventions from NHS TTad services. The statements were developed by SN and CF following RAND/UCLA methodology (e.g., appropriateness, necessity) and discussed, reviewed and refined with the research team and the PPI co‐applicant. A total of 29 statements were categorised into seven sections for patients (e.g., patient knowledge and engagement with relapse prevention) (Supporting Information 1: Appendix A) and an additional section with three further statements was added for professionals/key stakeholders (i.e., practitioner training before and after qualification) (Supporting Information 2: Appendix B). All statements were rated in phase 2.

### 3.2. Phase 2: Stakeholder Engagement via a Consensus‐Building Process

The results are structured to report on findings from the patient workshops first and then on professionals/key stakeholders.

## 4. Patients

### 4.1. Demographic Details

For the first workshop, eight patients expressed interest in participating, completed the first round of voting, and confirmed attendance. Five of these attended the first workshop. For the second workshop, four patients expressed interest in participating and confirmed attendance, of which three completed the first round of voting and attended the workshop.

For the final sample (*n* = 8), there were five females and three males, aged between 22 and 66 years, and all identified as straight/heterosexual, with two self‐reporting a disability. Additionally, three participants reported scores within the clinical range for the PHQ‐9/GAD‐7. Further demographic details are provided in Table 1.

**Table 1:** Summary of patient demographics and scores on routine outcome measures across both workshops and phases.

ID	Service	Age (years)	Relationship status	Ethnicity	Employment status	Religious background	Highest level of education	Routine outcome measure scores prior to workshop		
								PHQ‐9	GAD‐7	WSAS
Workshop 1										
PA1	B	49	Married	White	Not employed but not seeking work for some other reason	Christian	Master’s degree	0	0	0
PA3	B	66	Married	White	Retired	Agnostic	National certificate or diploma	4	5	6
PA5	A	33	Cohabiting	White European	Employed full time	Atheist	Degree (first/ordinary)	5	3	6
PA8	D	41	Married	Indian	Employed full time	Hindu	BTec certificate	5	13	34
P10 ^∗^	D	22	Single	White	Full time student	Agnostic	Degree (first/ordinary)	1	6	3
Workshop 2										
P11	C	66	Married	White	Retired	Agnostic	Degree (first/ordinary)	5	7	17
P12	C	42	Single	White	Self‐employed	Agnostic	NVQ/SVQ Levels 1–3	8	13	6
P13	D	33	Married	Other White— east European	Not employed but not seeking work for other reason	Agnostic	Don’t know	9	9	16
Phase 3										
P10 ^∗^	D	22	Single	White	Full time student	Agnostic	Degree (first/ordinary)	1	6	3
PAT01 ^∗∗^	B	62	Divorced	White	Employed full time	Atheist	PhD	3	1	19
PAT05 ^∗∗^	B	58	Other— legally separated	White	Other—retired but volunteering	Christian	GCE A/AS Level or Scottish higher	4	9	7

*Note:* ^∗^ = same participant,  ^∗∗^ = participant who took part in phase 1.

Abbreviations: GAD‐7, generalised anxiety disorder‐7 [31]; PHQ‐9, patient health questionnaire‐9 [30]; WSAS, work and social adjustment scale [33].

Supporting Information 3: File 1 presents the ratings per participant for rounds 1, 2 and 3 across patient workshops. Rankings of statements are presented in Table 2.

**Table 2:** Rankings of statements across four workshops.

Statements	Patient rankings		Professional/key stakeholder rankings	
	Workshop 1 (*n* = 5) (ranked out of 4)	Workshop 2 (*n* = 3) (ranked out of 17)	Workshop 1 (*n* = 5) (ranked out of 10)	Workshop 2 (*n* = 5) (ranked out of 18)
It is appropriate and necessary…				
Section 1. Service level engagement to relapse prevention				
To support recovery as part of routine care after the patient has reached recovery threshold	—	1	4	1
To provide patients with a consolidation/maintenance period after reaching the recovery threshold	—	2	3	4
That patients receive or have access to materials/resources used during sessions after reaching the recovery threshold	2	—	6	2
That patients have access to new materials/resources after reaching the recovery threshold which have not been used during sessions ^∗^	—	—	—	—
Section 2. Monitoring recovery after reaching the recovery threshold	—	—	—	—
To monitor clinical recovery after reaching the recovery threshold (using routine outcome measures including: PHQ‐9, GAD‐7 and WSAS)	—	12	—	—
To assess personal recovery after reaching the recovery threshold	—	10	1	6
That the same person who delivered treatment checks in with the patient after reaching the recovery threshold to monitor recovery ^∗^	—	—	—	—
That someone from NHS talking therapies services irrespective of whether they delivered treatment checks in with patients reaching the recovery threshold to monitor recovery	1	4	—	—
Section 3. External support after reaching the recovery threshold				
To involve social networks (friends, family and colleagues) in relapse prevention planning after reaching the recovery threshold	—	—	—	9
To involve the GP or other healthcare professionals outside of talking therapies services in relapse prevention planning after reaching the recovery threshold ^∗^	—	—	—	—
That the talking Therapies services provide INITIAL contact with external services that they signpost patients after reaching the recovery threshold, to address other needs	—	7	—	—
That talking Therapies services collaborate and communicate with local services in the health sector, including GPS to provide care to patients after reaching the recovery threshold	—	11	—	16
Section 4. Additional roles within TT services	—	—	—	—
To include patient representatives within NHS TT services to emphasise the importance of relapse prevention	—	—	—	14
To develop a specific role within NHS TT services for relapse prevention after patients reached the recovery threshold	3	15	—	—
Section 5. Recommendations to maintain progress/wellbeing after reaching the recovery threshold				
To provide refresher/booster courses for patients after reaching the recovery threshold to recap on treatment content	—	—	—	17
To have specific information in the NHS TT website for patients reaching the recovery threshold, including information regarding local resources/online resources, links to external support services, preventing relapse and so on	—	8	—	10
That the NHS TT services provide a 24‐h helpline for patients to connect with for a quick consultation regarding how to handle a particular situation causing symptoms of their anxiety/depression to resurface ^∗^	—	—	—	—
To provide patients after reaching the recovery threshold with access to a patient online forum, moderated by a qualified professional within the NHS TT service ^∗^	—	—	—	—
To connect two patients after reaching the recovery threshold with similar demographics and background to prevent relapse (i.e., a buddy support system) ^∗^	—	—	—	—
For patients after reaching the recovery threshold to access face‐to‐face support groups following end of treatment in talking therapy services ^∗^	—	—	—	—
Section 6. Awareness of guidelines and recommendations for relapse prevention				
That university training for PWPs captures recent policies, guidelines and recommendations surrounding relapse prevention	—	17	5	11
For clinical academics delivering university training for PWPs to be aware of up‐to‐date relapse prevention resources	—	13	2	3
That NHS TT staff delivering and supporting Step 2 treatment are knowledgeable about policies, guidelines and recommendations surrounding relapse prevention	—	14	7	5
For NHS TT staff delivering and supporting Step 2 treatment to be aware of up‐to‐date relapse prevention resources	—	6	8	12
For GPs and other external healthcare providers to be familiar with guidelines, policies and recommendations regarding relapse prevention in NHS TT services	—	16	—	18
Section 7. Expanding on training and ongoing discussions on relapse prevention (only applicable to professionals/key stakeholders)				
That university training for PWPs expands on relapse prevention	—	—	—	15
To provide continued professional development opportunities to for PWPs following qualification to develop further knowledge and skills on relapse prevention	—	—	10	—
That relapse prevention is discussed during clinical supervision	—	—	9	8
Section 8. Patient knowledge and engagement with relapse prevention				
That patients know the difference between a lapse and a relapse after reaching the recovery threshold	—	3	—	7
That patients’ regularly check in with themselves following treatment by recording/noting their mood	—	5	—	13
That patients are knowledgeable about the current process when returning to service	4	9	—	—
For NHS TT services to establish an independent route for patients reaching the recovery threshold, to return to service ^∗^	—	—	—	—

*Note:* — = statement not ranked,  ^∗^ statements not ranked by any of the four workshops.

### 4.2. Workshop 1 (*n* = 5)

For round 1, seven statements were rated in agreement and 22 in disagreement across participants and were discussed. The discussion focused on five of the seven sections of the form in which participants reflected on their ratings and experiences. In addition, the conversation clarified understanding of terminology used, such as differences between clinical and personal recovery and lapse versus relapse. Details of the moderated discussion, including illustrative quotes, are presented in Supporting Information 4: File 2.

During the second round, participants re‐rated all statements, from which 15 were rated in agreement, corresponding to awareness of guidelines and recommendations for relapse prevention. Statements rated in disagreement were related to accessing new resources after treatment, external support after reaching recovery, and recommendations to maintain progress after recovery.

For the final round, of the 15 statements rated in agreement, four were deemed necessary across all participants (Supporting Information 3: File 1) and were then ranked to identify priorities (Table 2).

### 4.3. Workshop 2 (*n* = 3)

For round 1, 18 statements were rated in agreement and 11 in disagreement, which were discussed. Statements rated in disagreement concerned external support following recovery and recommendations to maintain progress after reaching the recovery threshold. Similarly to the first patient workshop, the discussions focused on clarifying definitions and statements, and participants reported on their own experiences concerning some statements. Supporting Information 5: File 3 includes details of the discussion.

In round 2, one of the 29 statements was rated as not appropriate and in disagreement. This statement considered having the same person delivering treatment to monitor patient recovery over time.

For round 3, 17 statements were identified across all participants as necessary and ranked to identify priorities.

Supporting Information 3: File 1 presents the ratings per participant for rounds 1, 2 and 3 across patient workshops. Rankings of priorities are presented in Table 2.

## 5. Professionals and Key Stakeholders

### 5.1. Demographic Details

For the workshops, 35 professionals/key stakeholders expressed interest by contacting the research team. For the first workshop, nine participants agreed to participate on the scheduled day/time, and five attended. For the second workshop, five agreed to participate and attended. All participants completed the first round of voting prior to the workshop.

Across workshops, 10 professionals/key stakeholders participated, comprising nine females, aged between 20 and 65 years, all of whom self‐reported as straight or heterosexual, and nine of whom were of a white ethnic background. Participants comprised three clinical academics, two qualified PWPs, one senior PWP, one TTad team manager, one TTad lead counsellor, one researcher and one TTad screening team lead. See Table 3 for further demographic details.

**Table 3:** Professionals/key stakeholders’ demographic details across workshops and phases.

ID	Age (years)	Duration in current role (years)	Duration working in mental health (years)	When do they discuss relapse prevention?	Approach to relapse prevention	Advise provided to patients if they relapse following treatment
Workshop 1						
PR2	65	>5	>20	N/A	N/A	*‘Notice changes and contact either GP or self-refer. Some ad hoc advice may be given but this is not systematic’*
PR4 ^∗^	41	2 > 5	2 > 5	Other— throughout the sessions as necessary	*At the beginning and end of session* (*like sessions 5 and 6*) *and sometimes in between*	*‘Aim to use techniques they have acquired but if this isn’t successful*, *refer back into the service and/or seek support, for example, from a mental health line’*
PR5	40	2 > 5	10 > 20	Before the final session	*I’ll touch on it at the start when explaining how sessions work*, *then as we come to the end talk about completing a plan so they have something to refer back to when sessions end*.	*Check over the plan we’ve completed try to embed the things they were doing when they felt well*, *talk to family*, *but they can contact GP or come back to the service if after 6 months*
PR6 ^∗^	30	2 > 5	2 > 5	At the end of the final session	*Conversation*	*Re-refer*
PR7	45	1 > 2	>20	Before the final session	*Also discussing this when we learn something new and then again as we plan for ending – I utilise a template and complete this either with the client or the client might want to complete it as homework*	*Part of the maintenance plan is about identifying triggers*, *early warning signs and red flags— alongside this is what the client can do if any of these happen—what they can do*, *how others can help*, *who these other people are and so on*.
Workshop 2						
PR1	27	2 > 5	1 > 2	Before the final session	*No longer in practice—discussing with patients how to continue implementing techniques*, *reflecting on learning and progress*, *making notes to support recovery that they can revisit*, *normalising lapses*, *developing action plans*	*Re-refer to service. Re-visit materials from therapy. Draw on helpful coping strategies. Be kind to oneself*
PR9	45	2 > 5	>20	Before the final session	*Staying well protocol*	*Revisit their techniques and then re-refer if these are not successful*
PR10 ^∗^	57	>5	10 > 20	Other: when discharged patients contact my service in need of help	*Due to the unplanned nature of most of my patient contacts this tends to be ad hoc*	*Follow their relapse prevention plan in the first instance*
PR13	20	6 months >	1 > 2	N/A	*N/A*	*Re-engage with therapy* (*even if this is a brief return*), *Seek support*, *that is*, *family*, *friends*, *clinicians*, *Revisit coping strategies they learnt throughout treatment*, *with clinician re-evaluate underlying factors*, *that is*, *complete any necessary lifestyle changes and so on*.
PR14	44	>5	>20	Before the final session	*WhenIwas working clinically*, *usually at the penultimate session would discuss relapse prevention and provide written information/self-help workbook*	*To re-refer to the service*
Phase 3						
PR10 ^∗^	57	> 5	10 > 20	When discharged patients contact my service in need of help	Due to the unplanned nature of most of my patient contacts this tends to be ad hoc	*Follow their relapse prevention plan in the first instance*
PR4 ^∗^	41	2 < 5	2 < 5	Other – throughout the sessions as necessary	At the beginning and end of session (like sessions 5 and 6) and sometimes in between	*Aim to use techniques they have acquired but if this isn’t successful*, *refer back into the service and/or seek support for example from a mental health line*
PR6 ^∗^	30	2 < 5	2 < 5	At the end of the final session	Conversation	*Re-refer*
PR02 ^∗∗^	27	6 months <1	2 < 5	At the start of the final session	Encourage patients to continue to practice skills and techniques to feel the continued benefit and prevent relapse, educate patients on the difference between lapse and relapse, that is, having a bad day/week vs. relapse, educate on identifying signs of relapse and what to do if they notice signs	Speak to friends/family, re‐refer back in services, engage with the worksheets used in previous treatment

*Note:* ^∗^ = the same ID corresponds to the same person taking part in phase 3,  ^∗∗^ = participant from phase 1. The Italicised text indicates direct responses/quotations from participants.

Supporting Information 6: File 4 presents ratings across professional workshops for round 1, round 2 and round 3. Rankings of statements are presented in Table 2.

### 5.2. Workshop 1 (*n* = 5)

For round 1, 15 statements were rated in agreement, and 17 statements showed disagreement and were discussed prior to the second round of voting. Statements rated in disagreement were related to external support and recommendations to maintain progress. The discussion helped clarify statements’ vagueness, misunderstanding of language, and to contextualise participant’s neutral responses, which were contingent upon individual patient preferences. Details of the moderated discussion, including illustrative quotes, are presented in Supporting Information 7: File 5.

For round 2, all participants agreed on the appropriateness of 20 statements within the following three categories: (1) service level engagement to relapse prevention, (2) awareness of guidelines and recommendations for relapse prevention and (3) relapse prevention training. Twelve statements were rated in disagreement, including statements concerning external support and relapse prevention recommendations.

For the final round, of the 20 statements rated, 10 were agreed across all participants to be necessary (Supporting Information 6: File 4) and then ranked for priorities (Table 2).

### 5.3. Workshop 2 (*n* = 5)

For round 1, 17 statements were rated in agreement, and 15 showed disagreements and were discussed. Statements rated in disagreement concerned monitoring recovery, external support and recommendations to support recovery. The discussion centred around five of the eight sections of the form, focussing on potential benefits and challenges to implementing the statements within routine practice. Discussions clarified differences between what is appropriate and needed to support long‐term recovery and what is feasible to implement as stages within the intervention development process. Moreover, participants shared personal experiences related to the subject matter of each statement, which enriched understanding of people’s thinking, experiences and scores. Details of the discussion are presented in Supporting Information 8: File 6.

For round 2, participants agreed on 22 statements, which fall within the same three categories as for participants in workshop 1: (1) service level engagement to relapse prevention, (2) awareness of guidelines and recommendations for relapse prevention and (3) relapse prevention training.

For round 3, 18 of the 22 statements rated were deemed necessary across all participants and were ranked to identify priorities.

### 5.4. Results Within and Across Group Workshops

Patients rated 29 statements. Patients participating in the first workshop identified four statements as appropriate and necessary, and those participating in the second workshop identified 17. The discrepancy in numbers could be explained by people in the first workshop being influenced by what is feasible and achievable in the short term in an NHS‐constrained setting. The second group were encouraged not to limit their thinking to implementation challenges, as that was to be addressed later.

Across patient workshops, three statements identified as appropriate and necessary overlapped (Supporting Information 3: File 1). Table 2 shows the ranking for priorities.

Professionals/key stakeholders rated 32 statements. Those participating in the first workshop identified 10 statements as appropriate and necessary, and 18 statements were identified in the second workshop. Nine statements overlapped across professionals/key stakeholder workshops (Table 2).

### 5.5. Findings Across the Four Workshops

Across the four workshops, participants identified 24 solutions as essential for supporting long‐term wellbeing and preventing relapse. Seven of those overlapped between the second patient workshop and both professionals/key stakeholder workshops, focusing on supporting recovery post‐treatment, increasing guidelines awareness and promoting relapse prevention discussions among professionals. The first patient workshop and both professional workshops also converged on the importance of patients having ongoing access to treatment materials after achieving recovery.

Importantly, patients and professionals each identified unique priority areas, with some solutions endorsed exclusively by one group. This divergence highlights meaningful differences in how relapse prevention needs are perceived.

### 5.6. Patient‐Only Prioritised Solutions

• Regular monitoring of clinical recovery using treatment measures (e.g., PHQ‐9, GAD‐7 and WSAS).
• Ongoing wellbeing monitoring by NHS TTad personnel, regardless of prior treatment involvement.
• Creation of a dedicated role within NHS TTad to support long‐term recovery.
• Facilitating initial contact with external services, with patient consent, to aid ongoing wellbeing.
• Clear communication to patients about processes for regaining service access if needed.

### 5.7. Professional‐Only Prioritised Solutions

• Incorporation of relapse prevention discussions into clinical supervision.
• Continued professional development (CPD) opportunities post‐qualification to enhance relapse prevention skills among PWPs.
• Integration of relapse prevention training within university curricula for PWPs.
• Provision of refresher or booster sessions for patients after reaching recovery thresholds to reinforce learning.
• Inclusion of patient representatives in NHS TTad services to advocate for relapse prevention.
• Encouraging discussion of social network involvement (friends, family and colleagues) to sustain treatment benefits.

### 5.8. Smaller Sustained Group Work Between Phases With Members of the PPI Group

A smaller, sustained group involving close collaboration between three PPI members, the PPI co‐applicant, and the research team met between phases to review the solutions identified in phase 2 and complete preparatory work for phase 3. This collaborative group refined the clarity of the proposed solutions, explored how to present them in an accessible and user‐friendly format, and agreed on the structure and content of the final workshop. Through this article, it became clear that solutions addressed priorities across multiple levels, including patient care, professional practice, training curricula and policy. As a result, the original 24 solutions were synthesised into 13 broader, evidence‐informed recommendations (Table 4). The group also emphasised the need to translate each recommendation into actionable strategies and practical examples to support implementation. These were further developed and explored during phase 3.

**Table 4:** Details of 13 recommendations and proposed suggestions based upon output from smaller sustained group work.

Recommendation	Examples of suggestions to address recommendations
1. Create a dedicated section on the NHS talking therapies service website with up‐to‐date information on local services and online resources to support long‐term recovery	Develop a recovery resource hub or webpage for patients who have reached the recovery threshold, including: (1) Key terminology (e.g., lapse vs. relapse) (2) A step‐by‐step guide for returning to the service (text or video/animation) (3) Referral links and information on local services (4) Access to commonly used treatment resources (e.g., staying‐well booklets)
Discuss and agree on procedures to monitor patients who have achieved clinical recovery threshold over time	Option 1: The same practitioner schedules follow‐up check‐ins after the treatment Option 2: A different practitioner, who is familiar with the patient’s history, schedules follow‐up check‐ins after the treatment Option 3: Create a new role/team dedicated to maintenance and relapse prevention Option 4: Services to appoint a recovery champion to lead relapse prevention strategies throughout the therapeutic process and within the service. Option 5: Services may consider offering group refresher/booster sessions post‐recovery
Ensure staff are aware of and collaborate with local services to support long‐term recovery and to discuss these with patients	(1) Ensuring staff are knowledgeable about the local services in the area which target recovery, for example, recovery colleges (2) With patient consent, practitioners may contact external services directly and share referral information (3) Explain self‐referral pathways for external services to (4) Assist patients with completing any online referral forms for external services at the end of treatment
Assess clinical recovery after reaching the recovery threshold via the same routine outcome measures used during treatment	(1) Assess clinical recovery during follow‐up check‐ins using standard outcome measures (2) Assess clinical recovery through digital tools that incorporate ROMs, such as: • Apple health *mental health questionnaire* (Phq‐9 and GAD‐7) • The *Paddle therapy support app* (rollout planned for late 2025) • *Sorted: mental health app* (NHS digital accredited app)
Assess and monitor personal recovery after achieving clinical recovery	(1) Tailor treatment towards achieving individual personal gaols; and/or (2) Use established personal recovery measures/questionnaires
Ensure sufficient time in clinical supervision for relapse prevention and long‐term recovery discussions	Update the IAPT supervision guidance (last updated 2011) to include expectations for relapse prevention discussions
Ensure patients retain access to resources used during treatment after achieving recovery	(1) Provide patients with copies of standardised relapse prevention material used during treatment (2) Encourage patients to save emailed material on their preferred devices/folders (3) Promote use of apps that store treatment information for future access
Ensure patients understand current procedures for returning to the service after recovery	(1) Discuss return‐to‐service procedures before ending treatment and (2) Include clear information in the discharge letter
Encourage patients to self‐monitor their mood, thoughts and behaviours post‐treatment	Continue using a journal/diary or digital self‐monitoring tools as recommended during treatment
Discuss involvement of patients’ support networks in their staying‐well plan	Encourage patients to discuss their mental health with their support networks and identify ways others can support their wellbeing
Appoint patient representatives within NHS talking therapies to collaborate with professionals, and ensure patient voices shape post‐recovery support	Invite patients to join advisory groups or similar initiatives to share experiences and enhance service quality
Ensure national policy supports ongoing care after achieving clinical recovery from low‐intensity NHS talking therapies	Incorporate guidance in the talking therapies manual regarding follow‐up sessions 3 to 6 months post‐treatment. Address the ‘Revolving Door Phenomena’ [10] and reduce treatment return [34]
Ensure university training programmes include up‐to‐date relapse prevention policies, guidelines and resources	(1) Update teaching sessions to reflect current relapse prevention policies/guidelines (2) Develop a nationwide directory of relapse‐prevention resources (3) Disseminate updates within services (e.g., emails, meetings and supervisory sessions) (4) Provide relapse prevention CPD to ensure professionals are prepared to support long‐term recovery

*Note:* The Italicised text indicates direct responses/quotations from participants.

### 5.9. Phase 3: Shared Decision‐Making With Stakeholders

The shared decision‐making workshop included three patients, four professionals/key stakeholders and the research team. The research team included the PPI co‐applicant, the principal investigator and co‐principal investigator, two research collaborators and the research assistant.

Patients comprised two males and one female, aged between 22 and 68 years, with one reporting as having a disability. All patients self‐reported as straight or heterosexual (see Table 1 for demographic details). All professionals/key stakeholders were female, aged between 27 and 57 years, self‐reported as straight or heterosexual, had a postgraduate certificate, one considered themselves to have a disability, and one was of a White and Asian ethnicity (see Table 3 for further demographic details).

During the final workshop, a set of evidence‐based, stakeholder‐informed recommendations was refined to enhance post‐treatment mental health support following low‐intensity interventions.

Discussions in the final phase of the study revealed that the 13 stakeholder‐informed recommendations spanned multiple levels of implementation, including individual patients, practitioners, service providers, training institutions and policy stakeholders. Importantly, the recommendations varied in the time, effort and resources required for execution, ranging from low‐cost, easily implementable changes to more resource‐intensive, systemic reforms. To support sustained recovery, implementation should be accompanied by structured monitoring and evaluation.

For patients, recommendations emphasised empowering patients to self‐monitor mood, thoughts and behaviours post‐recovery, maintain access to therapy materials, engage with supportive social networks and understand procedures for returning to services if needed. Patients are also encouraged to involve personal networks in their staying‐well plans.

For practitioners, recommendations emphasised collaborative relapse prevention planning, setting clear next steps to maintain gains and mitigate relapse risk, allocating sufficient time during supervision to discuss long‐term recovery, and monitoring both clinical and personal recovery using routine outcome measures. Practitioners are also encouraged to foster awareness of and collaboration with local providers supporting ongoing wellbeing.

For services, recommendations included establishing formal post‐treatment monitoring protocols, appointing patient representatives to ensure service‐user voices inform monitoring and support, maintaining partnerships with community organisations and providing up‐to‐date online resources through NHS TTad websites to support long‐term recovery.

Recommendations for university and professional training included incorporating current policies, guidelines and relapse prevention resources into pre‐and post‐qualification curricula to ensure practitioners are equipped to support sustained recovery.

Recommendations for commissioners and policymakers emphasised embedding relapse prevention within workforce training, securing sustainable funding to support long‐term recovery pathways, and developing a compelling business case to ensure integration and sustainability of these initiatives.

Together, these recommendations provide a comprehensive framework spanning patient self‐management, professional practice, service processes, educational curricula and national policy. They offer actionable guidance for supporting long‐term recovery, while recognising that implementation will require tailored planning, allocation of resources and ongoing evaluation to ensure effectiveness.

## 6. Discussion

This study used a multiphase participatory design process to synthesise existing evidence and, in collaboration with stakeholders, co‐develop informed, evidence‐based solutions to support sustained mental health following low‐intensity treatment for anxiety and depression. Through evidence review, stakeholder consensus‐building and co‐production of recommendations, the findings offer a foundation for strengthening relapse prevention strategies and post‐treatment care within NHS TT (TTad) services.

Consistent with prior research [8, 9, 14], this study reinforces the need to move beyond short‐term symptom reduction and to focus on long‐term mental health maintenance. The stakeholder‐informed recommendations emphasise the importance of accessible, tailored resources that align with individual needs post‐discharge. These findings support the argument that recovery is not a static endpoint but a dynamic process requiring ongoing attention and support. Furthermore, the approach to recovery should be extended beyond symptom remission or clinical recovery alone. The CHIME framework—comprising connectedness, hope and optimism, identity, meaning in life and empowerment—highlights the multidimensional nature of personal recovery. This distinction is particularly relevant in the context of relapse prevention, where traditional models often focus narrowly on reducing symptom re‐emergence. By contrast, the CHIME framework provides a valuable lens through which to understand and address the psychosocial drivers of sustained wellbeing. For instance, feelings of isolation, loss of purpose or diminished self‐worth may not register as clinical deterioration but can significantly undermine recovery and increase vulnerability to relapse. Integrating CHIME‐informed principles into relapse prevention models ensures that support interventions address both clinical and personal dimensions of recovery. By aligning relapse prevention efforts with personal recovery frameworks, mental health services can provide more holistic, person‐centred care that supports lasting change and improved quality of life.

However, implementing innovations in routine practice remains challenging, especially within a stretched NHS context. As evidenced in implementation science [21], service transformation often falters due to system‐wide barriers, including staff shortages, rising demand and limited financial resources [35]. Consequently, sustainable change must extend beyond individual‐level interventions to encompass systemic and policy‐level reform. At the policy level, these findings highlight the need to reframe how mental health recovery, relapse prevention and long‐term wellbeing are positioned within NHS TTad services. Presently, outcome monitoring primarily focuses on symptom reduction from treatment initiation to discharge [29]. Yet, the NHS long term plan [36] recognises the need for enhanced aftercare and recovery‐focused services, acknowledging that conditions such as anxiety and depression often follow a fluctuating course. Embedding relapse prevention and wellbeing maintenance into the fabric of service delivery would align practice with this broader policy vision. In summary, this study underscores the value of evidence‐informed, collaboratively developed approaches for supporting individuals beyond initial treatment. Addressing long‐term wellbeing and relapse prevention in NHS TTad services will require both service‐level innovation and a shift in how mental health recovery is conceptualised and supported within policy and practice.

## 7. Strengths, Limitations and Future Research

This study benefits from a robust methodological framework that integrated the perspectives of multiple stakeholders, including individuals with lived experience, alongside professionals from diverse roles and services across the North of England. Partnership with a dedicated PPI advisory group strengthened the relevance and patient‐centredness of the research, ensuring that findings and recommendations were informed by both experiential and professional insights.

Despite purposive sampling to ensure variation across roles and service contexts, the sample was predominantly White. This limitation restricts the generalisability of findings to more ethnically diverse populations. This reflects the demographic profile of the current NHS TTad workforce and service user base, which remains skewed towards individuals of White ethnicity [37] and aligns with broader wider systemic, barriers identified for Black and minoritised ethnic groups (NHS Race and Health Observatory’s *Breaking Barriers for Better Health* report, 2023). Future research should therefore prioritise engaging more diverse populations to ensure cultural and contextual relevance. Additionally, literature suggests that some individuals who relapse following low‐intensity treatment may have benefitted from high‐intensity therapy [8, 9, 38]. While evaluating treatment allocation criteria was beyond the scope of this study, this has implications for understanding relapse risk and service planning, and future research could explore whether high‐intensity relapse‐prevention strategies can be adapted for low‐intensity contexts. Incorporating additional perspectives, such as those of carers, commissioners, and other professionals (e.g., high‐intensity practitioners or clinical psychologists), may also provide complementary insights and further strengthen the development of relapse‐prevention approaches.

Finally, the findings provide a foundation for feasibility studies to assess the acceptability, engagement and implementation of the co‐produced, evidence‐informed solutions. These studies will help redefine the approach, address key uncertainties and inform the design of a potential full‐scale randomised controlled trial aimed at sustaining recovery and preventing relapse.

## 8. Conclusion

Preventing relapse after low‐intensity treatment for anxiety and/or depression within NHS TT (TTad) services remains a significant challenge, highlighting the need for sustained support from professionals and services, leading to implications for future clinical practice, service design and policy development. This study co‐developed a set of evidence‐informed, stakeholder‐endorsed recommendations that lay the foundation for a relapse prevention model aimed at enhancing the long‐term effectiveness of treatment. These findings emphasise the need for sustainable, recovery‐focused approaches in routine care. Aligning relapse prevention strategies with a stepped care relapse prevention model allows for support to be proportionate, flexible and based on the individual’s needs. Integrating principles from the CHIME framework underscores the value of personal recovery—including connectedness, hope, identity, meaning and empowerment—beyond symptom reduction. Future work should focus on implementing the relapse prevention model recommendations and assessing its feasibility and impact in practice.

## Author Contributions

Saher Nawaz conducted a formal analysis of the data and was a major contributor to the manuscript. Penny Bee was involved in the funding acquisition and reviewed and edited the draft manuscript. Cintia Faija was involved in the funding acquisition, conceptualisation, methodology, project administration, supervision and a major contributor to the manuscript’s writing. All authors were involved in conceptualising the research.

## Funding

This study is funded by the National Institute for Health Research (NIHR) Research for Patient Benefit Programme (Grant: NIHR204037) led by Dr Cintia Faija and co‐led by Prof Penny Bee. The views expressed are those of the authors and not necessarily those of the NIHR or the Department of Health and Social Care.

## Disclosure

All authors read and approved the final manuscript. The funders had no role in study design, data collection, analysis or interpretation, decision to publish or preparation of the manuscript.

## Ethics Statement

Ethical approval was granted by the NHS Health Research Authority and Health and Care Research Wales (Reference: 23/NW/0109). Research governance was approved at all participating NHS TT sites.

## Consent

All participants provided informed consent and agreed to the following clause: I agree that any data collected may be included in anonymous form in publications/conference presentations.

## Conflicts of Interest

The authors declare no conflicts of interest.

## Supporting Information

Additional supporting information can be found online in the Supporting Information section.

## Supporting information


**Supporting Information 1** Appendix A: This file presents the complete list of 29 statements, categorised into 7 sections, that were rated by patients for appropriateness and necessity during the phase 2 workshops.

**Supporting Information 2** Appendix B: This file presents the complete list of 32 statements, rated on appropriateness and necessity by professionals and key stakeholders during the phase 2 workshops. This includes an additional category with 3 statements (26, 27, 28), which patients did not rate.

**Supporting Information 3** File 1: Patients’ ratings. This file presents two tables. The first table details individual patient ratings (out of 9) of the appropriateness of each statement across workshops 1 and 2. The second table details individual patient ratings per workshop for round 3 (out of 9) on the necessity of statements deemed appropriate and in agreement during round 2.

**Supporting Information 4** File 2: Patient WS1 Discussion. This file presents a table that displays details of the moderated discussion of statements rated in disagreement following the first round of voting for Patient Workshop 1. The table includes the key discussion points and some illustrative quotes from participants.

**Supporting Information 5** File 3: Patient WS2 Discussion. This file presents a table that displays details of the moderated discussion of statements rated in disagreement following the first round of voting for Patient Workshop 2. The table includes the key discussion points and some illustrative quotes from participants.

**Supporting Information 6** File 4: Prof Ratings. This file presents two tables. The first table details individual professional/key stakeholder ratings (out of 9) of the appropriateness of each statement across workshops 1 and 2. The second table details individual professional and key stakeholder ratings per workshop for round 3 (out of 9) on the necessity of statements deemed appropriate and in agreement during round 2.

**Supporting Information 7** File 5: Prof WS1 Discussion. This file presents a table that displays details of the moderated discussion of statements rated in disagreement following the first round of voting for the professional and key stakeholders’ workshop 1. The table includes the key discussion points and some illustrative quotes from participants.

**Supporting Information 8** File 6: Prof WS2 Discussion. This file presents a table that displays details of the moderated discussion of statements rated in disagreement following the first round of voting for the professional and key stakeholders’ workshop 2. The table includes the key discussion points and some illustrative quotes from participants.

## Data Availability

The data that supports the findings of this study are available in the Supporting Information of this article.

## References

[1] COVID-19 Mental Disorders Collaborators , Global Prevalence and Burden of Depressive and Anxiety Disorders in 204 countries and Territories in 2020 due to the COVID-19 Pandemic, The Lancet. (2021) 398, no. 10312, 1700–1712, 10.1016/S0140-6736(21)02143-7. PMC8500697 34634250

[2] WHO , Depressive Disorder (Depression), World Health Organisation, 2023, https://www.who.int/news-room/fact-sheets/detail/depression.

[3] Javaid S. F. , Hashim I. J. , Hashim M. J. , Stip E. , Samad M. A. , and Ahbabi A. A. , Epidemiology of Anxiety Disorders: Global Burden and Sociodemographic Associations, Middle East Current Psychiatry. (2023) 30, no. 1, 10.1186/s43045-023-00315-3, 44.

[4] Clark D. M. , Layard R. , Smithies R. , Richards D. A. , Suckling R. , and Wright B. , Improving Access to Psychological Therapy: Initial Evaluation of Two UK Demonstration Sites, Behaviour Research and Therapy. (2009) 47, no. 11, 910–920, 10.1016/j.brat.2009.07.010, 2-s2.0-70350526978. 19647230 PMC3111658

[5] NICE , Common Mental Health Problems: Identification and Pathways to Care, National Institute for Health and Care Excellence. (2011) https://www.nice.org.uk/guidance/cg123/chapter/recommendations.

[6] NHS , The National Collaborating Centre for Mental Health, NHS Talking Therapies for Anxiety and Depression Manual, 2024, March https://www.england.nhs.uk/wp-content/uploads/2018/06/nhs-talking-therapies-manual-v7.1-updated.pdf.

[7] NHS , NHS Talking Therapies, for Anxiety and Depression, Annual Reports, 2022–23, UK Government. (2024) https://digital.nhs.uk/data-and-information/publications/statistical/nhs-talking-therapies-for-anxiety-and-depression-annual-reports/2022-23.

[8] Ali S. , Rhodes L. , and Moreea O. , et al.How Durable is the Effect of Low Intensity CBT for Depression and Anxiety? Remission and Relapse in a Longitudinal Cohort Study, Behaviour Research and Therapy. (2017) 94, 1–8, 10.1016/j.brat.2017.04.006, 2-s2.0-85018513130. 28437680

[9] Delgadillo J. , Rhodes L. , and Moreea O. , et al.Relapse and Recurrence of Common Mental Health Problems After Low Intensity Cognitive Behavioural Therapy: The WYLOW Longitudinal Cohort Study, Psychotherapy and Psychosomatics. (2018) 87, no. 2, 116–117, 10.1159/000485386, 2-s2.0-85042349579. 29462816

[10] Roscoe J. , Has IAPT Become a Bit like Frankenstein’s Monster?, CBT Today. (2019) 47, no. 1, 16–17, https://insight.cumbria.ac.uk/id/eprint/6535/.

[11] Martin C. , Iqbal Z. , Airey N. D. , and Marks L. , Improving Access to Psychological Therapies (IAPT) has Potential but is not Sufficient: How Can It Better Meet the Range of Primary Care Mental Health Needs?, British Journal of Clinical Psychology. (2022) 61, no. 1, 157–174, 10.1111/bjc.12314. 34124792

[12] Kuyken W. , Warren F. C. , and Taylor R. S. , et al.Efficacy of Mindfulness-Based Cognitive Therapy in Prevention of Depressive Relapse: An Individual Patient Data Meta-Analysis From Randomized Trials, JAMA Psychiatry. (2016) 73, no. 6, 565–574, 10.1001/jamapsychiatry.2016.0076, 2-s2.0-84973325035. 27119968 PMC6640038

[13] Bruin E. , Muntingh A. D. , and Bourguignon E. M. , et al.Usage Intensity of a Relapse Prevention Program and Its Relation to Symptom Severity in Remitted Patients With Anxiety and Depression: Pre-Post Study, JMIR Mental Health. (2022) 9, no. 3, 10.2196/25441, e2544. PMC8968549 35293876

[14] Nawaz S. , Bee P. , Devaney H. , and Faija C. , Relapse Prevention Following Guided Self-Help for Common Health Problems: A Scoping Review, Cognitive Therapy and Research. (2024) 49, no. 1, 1–17, 10.1007/s10608-024-10520-x.

[15] Ward M. E. , De Brún A. , and Beirne D. , et al.Using Co-Design to Develop a Collective Leadership Intervention for Healthcare Teams to Improve Safety Culture, International Journal of Environmental Research and Public Health. (2018) 15, no. 6, 10.3390/ijerph15061182, 2-s2.0-85048253323, 1182. 29874883 PMC6025638

[16] Maurer M. , Mangrum R. , and Hilliard-Boone T. , et al.Understanding the Influence and Impact of Stakeholder Engagement in Patient-Centered Outcomes Research: A Qualitative Study, Journal of General Internal Medicine. (2022) 37, 6–13, 10.1007/s11606-021-07104-w. 35349017 PMC8993962

[17] Petkovic J. , Riddle A. , and Akl E. A. , et al.Protocol for the Development of Guidance for Stakeholder Engagement in Health and Healthcare Guideline Development and Implementation, Systematic Reviews. (2020) 9, no. 1, 10.1186/s13643-020-1272-5, 21. 32007104 PMC6995157

[18] Council Design , Framework for Innovation, 2019, https://www.designcouncil.org.uk/our-resources/framework-for-innovation/.

[19] Michie S. , Van Stralen M. M. , and West R. , The Behaviour Change Wheel: A New Method for Characterising and Designing Behaviour Change Interventions, Implementation Science. (2011) 6, no. 1, 1–12, 10.1186/1748-5908-6-42, 2-s2.0-79955006417. 21513547 PMC3096582

[20] Nilsen P. , Making Sense of Implementation Theories, Models and Frameworks, Implementation Science. (2015) 10, 10.1186/s13012-015-0242-0, 2-s2.0-84928389723, 53. 25895742 PMC4406164

[21] Moullin J. C. , Dickson K. S. , and Stadnick N. A. , et al.Ten Recommendations for Using Implementation Frameworks in Research and Practice, Implementation Science Communications. (2020) 1, no. 1, 1–12, 10.1186/s43058-020-00023-7. 32885199 PMC7427911

[22] Oliver H. , Thomas O. , and Neil R. , et al.A Longitudinal Study Combining the Double Diamond Framework and Behavior Change Wheel to Co-Create a Sedentary Behavior Intervention in Police Control Rooms, Journal of Public Health. (2024) 46, no. 3, 419–429, 10.1093/pubmed/fdae061. 38702850 PMC11358618

[23] Alicia O. , Croot L. , and Duncan E. , et al.Guidance on How to Develop Complex Interventions to Improve Health and Healthcare, BMJ Open. (2019) 9, no. 8, 10.1136/bmjopen-2019-029954, 2-s2.0-85070795293, e029954. PMC6701588 31420394

[24] Nawaz S. , Bee P. , and Faija C. , How to Maintain Recovery Following Low-Intensity Interventions for Anxiety and/or Depression? A Qualitative Exploration Through Perspectives of Professionals and Stakeholders, Journal of Affective Disorders. (2025) 372, 582–597, 10.1016/j.jad.2024.12.054. 39694332

[25] Fylan B. , Tomlinson J. , Raynor D. K. , and Silcock J. , Using Experience-Based Co-Design With Patients, Carers and Healthcare Professionals to Develop Theory-Based Interventions for Safer Medicines use, Research in Social and Administrative Pharmacy. (2021) 17, no. 12, 2127–2135, 10.1016/j.sapharm.2021.06.004. 34187746

[26] Campbell J. L. , Fletcher E. , and Abel G. , et al.Policies and Strategies to Retain and Support the Return of Experienced GPs in Direct Patient Care: The ReGROUP Mixed-Methods Study, Health and Social Care Delivery Research. (2019) 7, no. 14, 1–288. 30973692

[27] Jandhyala R. , Delphi, Non-RAND Modified Delphi, RAND/UCLA Appropriateness Method and a Novel Group Awareness and Consensus Methodology for Consensus Measurement: A Systematic Literature Review, Current Medical Research & Opinion. (2020) 36, no. 11, 1873–1887, 10.1080/03007995.2020.1816946. 32866051

[28] Fitch K. , Bernstein S. J. , Aguilar M. D. , Burnand B. , and LaCalle J. R. , The RAND/UCLA Appropriateness Method User’s Manual, 2001.

[29] Qualtrics , Qualtrics (Version 2020) [Software], 2020, Qualtrics, LLC, https://www.qualtrics.com.

[30] Kroenke K. , Spitzer R. L. , and Williams J. B. , The PHQ-9: Validity of a Brief Depression Severity Measure, Journal of General Internal Medicine. (2001) 16, no. 9, 606–613, 10.1046/j.1525-1497.2001.016009606.x, 2-s2.0-0034853189. 11556941 PMC1495268

[31] Spitzer R. L. , Kroenke K. , Williams J. B. , and Lowe B. , A Brief Measure for Assessing Generalized Anxiety Disorder: The GAD-7, Archives of Internal Medicine. (2006) 166, no. 10, 1092–1097, 10.1001/archinte.166.10.1092, 2-s2.0-33646815612. 16717171

[32] Released I. B. M. Corp , IBM SPSS Statistics for Windows, Version 29.0.2.0, 2023, IBM Corp.

[33] Mundt J. C. , Marks I. M. , Shear M. K. , and Greist J. M. , The Work and Social Adjustment Scale: A Simple Measure of Impairment in Functioning, British Journal of Psychiatry. (2002) 180, no. 5, 461–464, 10.1192/bjp.180.5.461, 2-s2.0-0036258012. 11983645

[34] Lorimer B. , Kellett S. , Giesemann J. , Lutz W. , and Delgadillo J. , An Investigation of Treatment Return After Psychological Therapy for Depression and Anxiety, Behavioural and Cognitive Psychotherapy. (2024) 52, no. 2, 149–162, 10.1017/S1352465823000322. 37563726

[35] Office National Audit , Progress in Improving Mental Health Services in England, UK Government. (2023) https://www.nao.org.uk/wp-content/uploads/2023/02/Progress-in-improving-mental-health-services-CS.pdf.

[36] NHS , The NHS Long Term Plan, UK Government, 2019, https://www.longtermplan.nhs.uk/publication/nhs-long-term-plan/.

[37] Health Education England , Health Education England NHS Talking Therapies for Anxiety and Depression Workforce Census, 2023, National Report. NHS https://www.hee.nhs.uk/sites/default/files/documents/HEE%20NHS%20Talking%20Therapies%20for%20Anxiety%20and%20Depression%20Workforce%20Census%202022%20-%20National%20Report.pdf.

[38] Thew G. R. , O’Reilly L. , and Andrews A. , et al.Blending Low-and High-Intensity Cognitive–behavioural Therapy in NHS Talking Therapies for Anxiety and Depression: Preliminary Evaluation, The British Journal of Psychiatry. (2025) 1–7, 10.1192/bjp.2025.10374. PMC7618677 40968478

[39] Papworth M. and Marrinan T. , Low Intensity Cognitive Behaviour Therapy, Psychology, 2018, Sage https://us.sagepub.com/sites/default/files/upm-assets/95439_book_item_95439.pdf.

