# Supplementary material for: Sustaining Recovery After Low‐Intensity Treatment for Anxiety and Depression in NHS Talking Therapies: A Multiphase Participatory and Consensus‐Building Study of Stakeholder Priorities and Recommendations
Source: Depress Anxiety. 2026 Jan 28;2026:9916526. doi: 10.1155/da/9916526 (PMC12852061; doi:10.1155/da/9916526)
Supplement: Supplementary file 1 — Supporting Information 1 Appendix A: This file presents the complete list of 29 statements, categorised into 7 sections, that were rated by patients for appropriateness and necessity during the phase 2 workshops. [file DA-2026-9916526-s008.docx]

**Appendix A**

***Phase 2 Statements for Patients***

**CO-IMPROVE Project Phase 2: Patients**

**Co-developing Improving Access to Psychological Therapies (IAPT) services to improve long-term benefits for patients with depression and anxiety**

We are interested in what you think we can do to maintain recovery over time and prevent the risk of relapse following patients’ discharge from low-intensity cognitive behavioural therapy treatment in talking therapies services.

Participant ID:

**Service Level:**

**Service level engagement to relapse prevention:**

1. How appropriate is it to support recovery as part of routine care after the patient has reached recovery threshold?
2. How appropriate is it to provide patients with a consolidation/maintenance period after reaching the recovery threshold?
3. How appropriate is it that patients receive or have access to materials/resources used during sessions after reaching the recovery threshold?
4. How appropriate is it that patients have access to new materials/resources after reaching the recovery threshold which have not been used during sessions?

**Monitoring Recovery after reaching the recovery threshold**

1. How appropriate is it to monitor clinical recovery after reaching the recovery threshold (using routine outcome measures including: PHQ-9, GAD-7, WSAS)?
2. How appropriate is it to assess personal recovery after reaching the recovery threshold?
3. How appropriate is it that the same person who delivered treatment checks in with the patient after reaching the recovery threshold to monitor recovery?
4. How appropriate is it that someone from NHS Talking Therapies services irrespective of whether they delivered treatment checks in with patients reaching the recovery threshold to monitor recovery?

**External Support after reaching the recovery threshold**

1. How appropriate is it to involve social networks (friends, family, colleagues) in relapse prevention planning after reaching the recovery threshold?
2. How appropriate is it to involve the GP or other healthcare professionals outside of NHS Talking Therapies services in relapse prevention planning after reaching the recovery threshold?
3. How appropriate is it that the NHS Talking Therapies services provide INITIAL contact with external services that they signpost patients after reaching the recovery threshold, to address other needs?
4. How appropriate is it that NHS Talking Therapies services collaborate and communicate with local services in the health sector including GPs to provide care to patients after reaching the recovery threshold?

**Additional Roles within TT services:**

1. How appropriate is it to include patient representatives within NHS Talking Therapies Services to emphasise the importance of relapse prevention?
2. How appropriate is it to develop a specific role within NHS Talking Therapies services for relapse prevention after patients reached the recovery threshold?

**Recommendations to maintain progress/wellbeing after reaching the recovery threshold**

1. How appropriate is it to provide refresher/booster courses for patients after reaching the recovery threshold to recap on treatment content?
2. How appropriate is it to have specific information in the NHS Talking Therapies website for patients reaching the recovery threshold including information regarding local resources/online resources, links to external support services, preventing relapse etc?
3. How appropriate is it that the NHS Talking Therapies services provide a 24-hour helpline for patients to connect with for a quick consultation regarding how to handle a particular situation causing symptoms of their anxiety/depression to resurface?
4. How appropriate is it to provide patients after reaching the recovery threshold with access to a patient online forum, moderated by a qualified professional within the NHS Talking Therapies service?
5. How appropriate is it for NHS Talking Therapies to connect two patients after reaching the recovery threshold with similar demographics and background to prevent relapse (i.e., a buddy support system)?
6. How appropriate is it for NHS Talking Therapies patients after reaching the recovery threshold to access face-to-face support groups following end of treatment in talking therapy services?

**Awareness of guidelines and recommendations for relapse prevention:**

1. How appropriate is it that university training for Psychological Wellbeing Practitioners captures recent policies, guidelines and recommendations surrounding relapse prevention?
2. How appropriate is it for clinical academics delivering university training for Psychological Well Being Practitioners to be aware of up-to-date relapse prevention resources?
3. How appropriate is it that NHS Talking Therapies staff delivering and supporting step 2 treatment are knowledgeable about policies, guidelines and recommendations surrounding relapse prevention?
4. How appropriate is it for NHS Talking Therapies staff delivering and supporting step 2 treatment to be aware of up-to-date relapse prevention resources?
5. How appropriate is it for GPs and other external healthcare providers to be familiar with guidelines, policies and recommendations regarding relapse prevention in Talking Therapies services?

**Patient knowledge and engagement with relapse prevention**

1. How appropriate is it that patients know the difference between a lapse and a relapse after reaching the recovery threshold?
2. How appropriate is it that patients’ regularly check in with themselves following treatment by recording/noting their mood and patterns/trends?
3. How appropriate is it that patients are knowledgeable about the current process when returning to service?
4. How appropriate is it for NHS Talking Therapies services to establish an independent route for patients reaching the recovery threshold, to return to service?
