# Supplementary material for: Sustaining Recovery After Low‐Intensity Treatment for Anxiety and Depression in NHS Talking Therapies: A Multiphase Participatory and Consensus‐Building Study of Stakeholder Priorities and Recommendations
Source: Depress Anxiety. 2026 Jan 28;2026:9916526. doi: 10.1155/da/9916526 (PMC12852061; doi:10.1155/da/9916526)
Supplement: Supplementary file 3 — Supporting Information 3 File 1: Patients’ ratings. This file presents two tables. The first table details individual patient ratings (out of 9) of the appropriateness of each statement across workshops 1 and 2. The second table details individual patient ratings per workshop for round 3 (out of 9) on the necessity of statements deemed appropriate and in agreement during round 2. [file DA-2026-9916526-s005.docx]

**Supplementary File 1.** *Ratings of statements for each round across both patient workshops.*

Round 1 and 2 Ratings

| **Statements** | **Round 1 ratings (1 - 9)** | | | | | | | | | | | **Round 2 Ratings (1 - 9)** | | | | | | | | |
| --- | --- | --- | --- | --- | --- | --- | --- | --- | --- | --- | --- | --- | --- | --- | --- | --- | --- | --- | --- | --- |
|  | **Workshop 1 (n=8)** | | | | | | | | **Workshop 2 (n=3)** | | | **Workshop 1 (n=5)** | | | | | **Workshop 2 (n=3)** | | | |
| ***How appropriate is it…*** | **PA1** | **PA2*** | **PA3** | **PA5** | **PA6*** | **PA8** | **PA9*** | **P10** | **P11** | **P12** | **P13** | **PA1** | **PA3** | **PA5** | **PA8** | **P10** | | **P11** | **P12** | **P13** |
| ***Section 1. Service level engagement to relapse prevention*** | | | | | | | | | | | | | | | | | | | | |
| To support recovery as part of routine care after the patient has reached recovery threshold? | 9 | 7 | 9 | 9 | 7 | 7 | 9 | 7 | 9 | 7 | 8 | 9 | 9 | 9 | 8 | 9 | | 9 | 8 | 9 |
| To provide patients with a consolidation/maintenance period after reaching the recovery threshold? | 9 | 9 | 9 | 9 | 7 | 8 | 9 | 9 | 9 | 7 | 9 | 9 | 9 | 9 | 9 | 9 | | 9 | 8 | 9 |
| That patients receive or have access to materials/resources used during sessions after reaching the recovery threshold? | 9 | 9 | 7 | 9 | 7 | 9 | 7 | 9 | 9 | 6 | 9 | 9 | 7 | 9 | 9 | 9 | | 9 | 9 | 9 |
| That patients have access to new materials/resources after reaching the recovery threshold which have not been used during sessions? | 6 | 5 | 7 | 9 | 5 | 6 | 9 | 7 | 9 | 9 | 8 | 6 | 7 | 9 | 5 | 8 | | 9 | 9 | 9 |
| **Section 2. Monitoring recovery after reaching the recovery threshold** | | | | | | | | | | | | | | | | | | | | |
| To monitor clinical recovery after reaching the recovery threshold (using routine outcome measures including: PHQ-9, GAD-7, WSAS)? | 8 | 9 | 5 | 9 | 7 | 7 | 6 | 7 | 7 | 9 | 8 | 8 | 5 | 6 | 8 | 8 | | 9 | 8 | 9 |
| To assess personal recovery after reaching the recovery threshold? | 8 | 9 | 7 | 9 | 7 | 7 | 6 | 7 | 9 | 9 | 8 | 8 | 7 | 9 | 9 | 9 | | 9 | 9 | 9 |
| That the same person who delivered treatment checks in with the patient after reaching the recovery threshold to monitor recovery? | 9 | 9 | 7 | 5 | 5 | 9 | 9 | 8 | 7 | 7 | 9 | 8 | 7 | 5 | 9 | 9 | | 5 | 7 | 9 |
| That someone from NHS TT services irrespective of whether they delivered treatment checks in with patients reaching the recovery threshold to monitor recovery? | 9 | 8 | 9 | 9 | 7 | 3 | 7 | 9 | 9 | 9 | 5 | 9 | 9 | 9 | 9 | 9 | | 9 | 8 | 8 |
| **Section 3. External support after reaching the recovery threshold** | | | | | | | | | | | | | | | | | | | | |
| To involve social networks (friends, family, colleagues) in relapse prevention planning after reaching the recovery threshold? | 8 | 4 | 5 | 7 | 8 | 8 | 5 | 8 | 7 | 9 | 5 | 8 | 5 | 6 | 9 | 7 | | 7 | 8 | 8 |
| To involve the GP or other healthcare professionals outside of NHS TT services in relapse prevention planning after reaching the recovery threshold? | 9 | 9 | 9 | 9 | 5 | 7 | 7 | 5 | 7 | 7 | 4 | 9 | 9 | 9 | 9 | 5 | | 8 | 8 | 9 |
| That the NHS TT services provide INITIAL contact with external services that they signpost patients after reaching the recovery threshold, to address other needs? | 9 | 5 | 5 | 7 | 6 | 8 | 8 | 7 | 7 | 7 | 5 | 9 | 5 | 9 | 9 | 7 | | 9 | 8 | 9 |
| That NHS TT services collaborate and communicate with local services in the health sector including GPS to provide care to patients after reaching the recovery threshold? | 9 | 9 | 5 | 9 | 5 | 6 | 7 | 7 | 8 | 8 | 5 | 9 | 9 | 9 | 8 | 6 | | 9 | 8 | 9 |
| **Section 4. Additional roles within TT services** | | | | | | | | | | | | | | | | | | | | |
| To include patient representatives within NHS TT Services to emphasise the importance of relapse prevention? | 8 | 9 | 7 | 8 | 6 | 8 | 9 | 9 | 9 | 9 | 6 | 8 | 8 | 8 | 9 | 9 | | 9 | 9 | 9 |
| To develop a specific role within NHS TT services for relapse prevention after patients reached the recovery threshold? | 8 | 9 | 6 | 9 | 7 | 8 | 7 | 9 | 9 | 9 | 9 | 8 | 8 | 9 | 9 | 9 | | 9 | 8 | 9 |
| **Section 5. Recommendations to maintain progress/wellbeing after reaching the recovery threshold** | | | | | | | | | | | | | | | | | | | | |
| To provide refresher/booster courses for patients after reaching the recovery threshold to recap on treatment content? | 9 | 9 | 6 | 9 | 5 | 8 | 9 | 9 | 9 | 9 | 9 | 9 | 6 | 9 | 5 | 9 | | 9 | 8 | 9 |
| To have specific information in the NHS TT website for patients reaching the recovery threshold including information regarding local resources/online resources, links to external support services, preventing relapse etc? | 9 | 9 | 9 | 9 | 7 | 5 | 9 | 9 | 9 | 9 | 8 | 9 | 9 | 9 | 5 | 9 | | 9 | 8 | 9 |
| That the NHS TT services provide a 24-hour helpline for patients to connect with for a quick consultation regarding how to handle a particular situation causing symptoms of their anxiety/depression to resurface? | 8 | 9 | 8 | 6 | 5 | 7 | 8 | 9 | 9 | 9 | 8 | 8 | 5 | 5 | 9 | 9 | | 9 | 8 | 9 |
| To provide patients after reaching the recovery threshold with access to a patient online forum, moderated by a qualified professional within the NHS TT service? | 7 | 5 | 5 | 7 | 5 | 5 | 7 | 8 | 9 | 9 | 9 | 8 | 5 | 4 | 9 | 8 | | 9 | 9 | 9 |
| To connect two patients after reaching the recovery threshold with similar demographics and background to prevent relapse (i.e., a buddy support system)? | 5 | 1 | 1 | 7 | 7 | 6 | 8 | 6 | 8 | 8 | 6 | 6 | 2 | 8 | 8 | 5 | | 7 | 8 | 9 |
| For patients after reaching the recovery threshold to access face-to-face support groups following end of treatment in talking therapy services? | 5 | 2 | 5 | 7 | 5 | 8 | 7 | 8 | 9 | 9 | 4 | 7 | 5 | 9 | 9 | 8 | | 9 | 9 | 9 |
| **Section 6. Awareness of guidelines and recommendations for relapse prevention:** | | | | | | | | | | | | | | | | | | | | |
| That university training for Psychological Wellbeing Practitioners captures recent policies, guidelines and recommendations surrounding relapse prevention? | 9 | 9 | 9 | 9 | 7 | 7 | 8 | 9 | 9 | 9 | 5 | 9 | 9 | 9 | 9 | 9 | | 9 | 9 | 9 |
| For clinical academics delivering university training for Psychological Wellbeing Practitioners to be aware of up-to-date relapse prevention resources? | 9 | 9 | 9 | 9 | 7 | 8 | 9 | 9 | 9 | 9 | 9 | 9 | 9 | 9 | 9 | 9 | | 9 | 9 | 9 |
| That NHS TT staff delivering and supporting step 2 treatment are knowledgeable about policies, guidelines and recommendations surrounding relapse prevention? | 9 | 9 | 9 | 9 | 7 | 8 | 8 | 9 | 9 | 9 | 7 | 9 | 9 | 9 | 9 | 9 | | 9 | 9 | 9 |
| For NHS TT staff delivering and supporting step 2 treatment to be aware of up-to-date relapse prevention resources? | 9 | 9 | 9 | 9 | 7 | 9 | 9 | 9 | 9 | 9 | 9 | 9 | 9 | 9 | 9 | 9 | | 9 | 9 | 9 |
| For GPs and other external healthcare providers to be familiar with guidelines, policies and recommendations regarding relapse prevention in NHS TT services? | 9 | 9 | 9 | 9 | 5 | 8 | 9 | 9 | 9 | 9 | 9 | 9 | 9 | 9 | 9 | 9 | | 9 | 9 | 9 |
| **Section 7. Patient knowledge and engagement with relapse prevention** | | | | | | | | | | | | | | | | | | | | |
| That patients know the difference between a lapse and a relapse after reaching the recovery threshold? | 9 | 9 | 6 | 9 | 5 | 9 | 9 | 7 | 9 | 6 | 9 | 9 | 8 | 9 | 9 | 7 | | 9 | 9 | 9 |
| That patients’ regularly check in with themselves following treatment by recording/noting their mood? | 9 | 9 | 8 | 9 | 5 | 9 | 8 | 9 | 8 | 9 | 9 | 9 | 8 | 9 | 9 | 9 | | 9 | 9 | 9 |
| That patients are knowledgeable about the current process when returning to service? | 8 | 9 | 5 | 9 | 5 | 8 | 9 | 9 | 9 | 9 | 8 | 9 | 9 | 9 | 9 | 9 | | 9 | 9 | 9 |
| For NHS TT services to establish an independent route for patients reaching the recovery threshold, to return to service? | 8 | 9 | 7 | 9 | 5 | 7 | 7 | 9 | 9 | 7 | 8 | 8 | 5 | 9 | 9 | 9 | | 9 | 8 | 9 |

*Note.* * = Participants completed round one prior to workshop but did not attend workshop.

**Round 3 Ratings**

| **Statements** | **Round 3 Ratings (1 - 9)** | | | | | | | | |
| --- | --- | --- | --- | --- | --- | --- | --- | --- | --- |
|  | **Workshop 1 (n=5)** | | | | | **Workshop 2 (n=3)** | | | |
| ***How necessary is it…*** | **PA1** | **PA3** | **PA5** | **PA8** | **P10** | | **P11** | **P12** | **P13** |
| ***Section 1. Service level engagement to relapse prevention*** |  |  |  |  |  | |  |  |  |
| To support recovery as part of routine care after the patient has reached recovery threshold? | 9 | 7 | 9 | 1 | 9 | | 8 | 8 | 9 |
| To provide patients with a consolidation/maintenance period after reaching the recovery threshold? | 9 | 7 | 9 | 1 | 9 | | 9 | 8 | 9 |
| That patients receive or have access to materials/resources used during sessions after reaching the recovery threshold? | 9 | 7 | 9 | 9 | 9 | | 6 | 9 | 9 |
| That patients have access to new materials/resources after reaching the recovery threshold which have not been used during sessions? | - | - | - | - | - | | 6 | 3 | 8 |
| **Section 2. Monitoring recovery after reaching the recovery threshold** |  |  |  |  |  | |  |  |  |
| To monitor clinical recovery after reaching the recovery threshold (using routine outcome measures including: PHQ-9, GAD-7, WSAS)? | - | - | - | - | - | | 7 | 9 | 7 |
| To assess personal recovery after reaching the recovery threshold? | 9 | 7 | 9 | 1 | 9 | | 9 | 9 | 7 |
| That the same person who delivered treatment checks in with the patient after reaching the recovery threshold to monitor recovery? | - | - | - | - | - | | - | - | - |
| That someone from NHS TT services irrespective of whether they delivered treatment checks in with patients reaching the recovery threshold to monitor recovery? | 9 | 7 | 9 | 9 | 9 | | 9 | 9 | 7 |
| **Section 3. External support after reaching the recovery threshold** |  |  |  |  |  | |  |  |  |
| To involve social networks (friends, family, colleagues) in relapse prevention planning after reaching the recovery threshold? | - | - | - | - | - | | 6 | 9 | 5 |
| To involve the GP or other healthcare professionals outside of NHS TT services in relapse prevention planning after reaching the recovery threshold? | - | - | - | - | - | | 9 | 9 | 6 |
| That the NHS TT services provide INITIAL contact with external services that they signpost patients after reaching the recovery threshold, to address other needs? | - | - | - | - | - | | 9 | 9 | 7 |
| That NHS TT services collaborate and communicate with local services in the health sector including GPS to provide care to patients after reaching the recovery threshold? | - | - | - | - | - | | 9 | 9 | 7 |
| **Section 4. Additional roles within TT services** |  |  |  |  |  | |  |  |  |
| To include patient representatives within NHS TT Services to emphasise the importance of relapse prevention? | 8 | 5 | 5 | 9 | 9 | | 6 | 9 | 9 |
| To develop a specific role within NHS TT services for relapse prevention after patients reached the recovery threshold? | 8 | 7 | 9 | 9 | 9 | | 7 | 9 | 9 |
| **Section 5. Recommendations to maintain progress/wellbeing after reaching the recovery threshold** |  |  |  |  |  | |  |  |  |
| To provide refresher/booster courses for patients after reaching the recovery threshold to recap on treatment content? | - | - | - | - | - | | 6 | 9 | 9 |
| To have specific information in the NHS TT website for patients reaching the recovery threshold including information regarding local resources/online resources, links to external support services, preventing relapse etc? | - | - | - | - | - | | 9 | 9 | 8 |
| That the NHS TT services provide a 24-hour helpline for patients to connect with for a quick consultation regarding how to handle a particular situation causing symptoms of their anxiety/depression to resurface? | - | - | - | - | - | | 5 | 8 | 8 |
| To provide patients after reaching the recovery threshold with access to a patient online forum, moderated by a qualified professional within the NHS TT service? | - | - | - | - | - | | 5 | 9 | 8 |
| To connect two patients after reaching the recovery threshold with similar demographics and background to prevent relapse (i.e., a buddy support system)? | - | - | - | - | - | | 5 | 8 | 5 |
| For patients after reaching the recovery threshold to access face-to-face support groups following end of treatment in talking therapy services? | - | - | - | - | - | | 8 | 9 | 5 |
| **Section 6. Awareness of guidelines and recommendations for relapse prevention:** |  |  |  |  |  | |  |  |  |
| That university training for Psychological Wellbeing Practitioners captures recent policies, guidelines and recommendations surrounding relapse prevention? | 9 | 8 | 7 | 1 | 9 | | 9 | 9 | 8 |
| For clinical academics delivering university training for Psychological Wellbeing Practitioners to be aware of up-to-date relapse prevention resources? | 9 | 8 | 9 | 1 | 9 | | 9 | 9 | 8 |
| That NHS TT staff delivering and supporting step 2 treatment are knowledgeable about policies, guidelines and recommendations surrounding relapse prevention? | 9 | 9 | 7 | 1 | 9 | | 9 | 9 | 8 |
| For NHS TT staff delivering and supporting step 2 treatment to be aware of up-to-date relapse prevention resources? | 9 | 9 | 9 | 1 | 9 | | 9 | 9 | 8 |
| For GPs and other external healthcare providers to be familiar with guidelines, policies and recommendations regarding relapse prevention in NHS TT services? | 9 | 9 | 9 | 1 | 9 | | 9 | 9 | 8 |
| **Section 7. Patient knowledge and engagement with relapse prevention** |  |  |  |  |  | |  |  |  |
| That patients know the difference between a lapse and a relapse after reaching the recovery threshold? | 8 | 7 | 6 | 9 | 8 | | 9 | 9 | 8 |
| That patients’ regularly check in with themselves following treatment by recording/noting their mood? | 8 | 6 | 8 | 9 | 9 | | 9 | 9 | 8 |
| That patients are knowledgeable about the current process when returning to service? | 9 | 7 | 9 | 9 | 9 | | 9 | 9 | 7 |
| For NHS TT services to establish an independent route for patients reaching the recovery threshold, to return to service? | - | - | - | - | - | | 5 | 9 | 7 |

Note. - = Not Rated.
