# Supplementary material for: Sustaining Recovery After Low‐Intensity Treatment for Anxiety and Depression in NHS Talking Therapies: A Multiphase Participatory and Consensus‐Building Study of Stakeholder Priorities and Recommendations
Source: Depress Anxiety. 2026 Jan 28;2026:9916526. doi: 10.1155/da/9916526 (PMC12852061; doi:10.1155/da/9916526)
Supplement: Supplementary file 4 — Supporting Information 4 File 2: Patient WS1 Discussion. This file presents a table that displays details of the moderated discussion of statements rated in disagreement following the first round of voting for Patient Workshop 1. The table includes the key discussion points and some illustrative quotes from participants. [file DA-2026-9916526-s004.docx]

**Supplementary File 2.**

*Patients Workshop 1: Overview of the moderated discussions of statements with disagreement following round 1 voting and illustrative quotes.*

| **Statement** | **Key discussion points** | **Quotes** |
| --- | --- | --- |
| **How appropriate is it …** |  |  |
| **Section 1:** Access to new material | | |
| 4. that patients have access to new material/resources after reaching the recovery threshold which have not been | It is important to consider the potential drawbacks when introducing new material to aid patients in their recovery journey. While providing additional support is beneficial, the lack of guidance in using the new materials following treatment could overwhelm patients and lead to overthinking about. Also, patients may not use the new material as they are familiar with and have found the material using during session as helpful for them. | I stated, neutral. This is a result of me thinking it could work either way. These new materials could be immensely helpful to the patient, they could help consolidate recovery. But, on the other hand, the patient could begin to overthink and doubt processes they've previously found very helpful, and there could almost be a clouding of their perspective… It could be immensely helpful, but it could also potentially seek to destabilize some present recovery mechanisms (P10)  the thing is that when we were during the sessions we worked with those resources, together with our, with our counsellor, with the practitioner. So I'm kind of hesitant right now thinking about receiving new resources after the treatment, and then, if I had no support 1^st^ of all, I might not. I might just not do anything, because, you know, nobody's there to check on me. And that's kind of my personal self-compliant issues. But also, maybe I wouldn't know how to process those new materials like if I don't have someone to talk them through with it. (PA5) |
| **Section 2:** Clinical and personal recovery | | |
| 5. to monitor clinical recovery after reaching the recovery threshold (using routine outcome measures including: PHQ-9, GAD-7, WSAS)? | Keeping track of your recovery through journaling is beneficial, but it can be challenging to maintain this habit. Therefore, it is helpful to have a way to consistently record and note down your moods and reactions to different situations over a long period of time. This can help in measuring your recovery progress and reinforcing positive behaviours during difficult times. While using questionnaires to measure clinical recovery has its benefits, it may not capture temporary stressors in your life, such as upcoming deadlines, which can affect your questionnaire responses. This makes it hard to distinguish between signs of potential relapse and challenges that might lead to a resurgence of symptoms. | ‘I've always found that writing a journal always helps in measuring how much you have recovered. But of course, I get lazy, and I lose the track of it and stop writing journal completely … But if there is a certain criteria that an individual can set for themselves, like in a given situation, how was my mood? Or how was my reaction? If someone is able to note it down, then I’m sure it will work, help for that individual to behave in a better or in an appropriate way what they consider for themselves in a similar situation that comes up in future.’ (PA9)  ‘In terms of the clinical recovery. I think it's I'm somewhat torn between the 2 sides. On one hand, I think it is an immensely valuable resource for measuring your levels of anxiety and your levels of depressive symptoms. And that understanding where you are along those lines is can spot potentially problematic signs of you entering a place of distress earlier on and can potentially direct you to resources earlier. However, I found some of the questions to be somewhat emphasised. There were, there were weeks when my stress level would be higher than standard, and that would be reflected in the questionnaire that were not specifically anything to do with my mental health. It was to do with circumstances in my own life. Potentially, you know, upcoming deadlines, medical appointments that would be considered stressful. So it's a valuable resource, I just wonder how we would differentiate between potentially stressful or difficult times within the individual patient's life and potentially problematic signs that could be more indicative of a relapse or entering a more difficult place.’ (P10) |
| 6. to assess personal recovery after reaching the recovery threshold? |  |  |
| **Section 2:** If anyone, who should be responsible for monitoring the patient afterwards (same practitioner or different practitioner from NHS talking Therapies) | | |
| 7. that the same person who delivered treatment checks in with the patient after reaching the recovery threshold to monitor recovery? | When determining who should check in with a patient, the preference is for the therapist who provided the treatment to do so. If this is not possible, as long as someone from TT checks in and has reviewed the patient notes to understand their history, that is better than no one checking in at all. | It doesn't matter to be the same practitioner. Anyone from the service. As long as you know there is you know, clear communication of the you know the patient's history essentially… I understand also that people move. They change jobs. They move places. So, it's a little bit unrealistic to expect to have the same practitioner. (PA5)  when you talk to the same person it feels that you start from where you left off. But when you talk to a totally different person it would kind of feel like. 1st of all, you don't feel 100% comfortable in in sharing probably what you have already shared with the person you took treatment from, but it would probably take some time before you can open up and be able to feel the same level of comfort. That's why I rated the way I did. (PA9) |
| 8. That someone from NHS Talking Therapies services irrespective of whether they delivered treatment checks in with patients reaching the recovery threshold to monitor recovery? |  |  |
| **Section 3: Support from personal networks and GPs / local services.** | | |
| 9. to involve social networks (friends, family, colleagues) in relapse prevention planning after reaching the recovery threshold? | Discussing the therapy content with family can encourage further insights, and their knowledge of the treatment would provide support after completion to help consolidate the learning.  Whilst friends and family were seen as appropriate to include as part of support networks, colleagues could be an issue due to confidentiality. Therefore, it is impotent to consider the Patients choice in such matters of who should know and to what extent they should be involved. | I talk everything that I go through with my wife, whether it's part of the therapy, or even the daily thing, and that actually helps quite a lot like if I have talked to the therapist. Whatever discussion we had, I go and discuss with my wife, and she adds on to that, so that actually doubly helps in in a way that I can. I can get more insight of whatever has been discussed. She motivates me in writing the journal, and things like that. So an involvement of a family, at least in my case, has a definite big advantage or a big boost in in the way I handle the recovery phase or any treatment. (PA9)  I think it needs to be balanced. Of course, friends and family is something that would generally be appropriate. But I'm also thinking alongside confidentiality. Once you get to, once you get to colleagues, people that would directly be working with you (P10)  I think it comes down to sort of. I guess that personal choice, isn't it? If it's something that is going to be helpful for that particular individual, and it's going to benefit them. (PA1) |
| 10. involve the GP or other healthcare professionals outside of NHS Talking Therapies services in relapse prevention planning after reaching the recovery threshold? |  |  |
| 12. that NHS Talking Therapies services collaborate with local services in the health sector including GPs to provide care to patients after reaching the recovery threshold? |  |  |
| **Section 3. NHS Talking Therapies services responsible for the initial contact with external services.** | | |
| 11. That the NHS Talking Therapies services provides INITIAL contact with external services that they signpost patients after reaching the recovery threshold, to address other needs. | The initial contact would enhance the credibility of the external service and help patients feel that their health is valued, providing a sense of community support.  It is important that this discussion is held with patients prior to the service contacting them to ensure they view the services as helpful and ensure they are playing an active role in their care. | ‘ I suppose, for me it would also make it feel more like these. Other services are kind of more official or more legit, you know, if they are directly contacted by the NHS services, you know, and then they reach out to me like it would feel more like. oh, yes, I'm being taken care of that. There are people that are actually, you know, part of the bigger community of practitioners, or you know, communities in general and even local communities that can help me.’ (PA5)  That it does give them a degree of legitimacy. And it makes you feel more comfortable with the services in question. I would add, as long as there had been some consultation with the patient prior to that initial contact being made by the services is fine. (P10) |
| **Section 5. Mediated support by NHS Talking Therapies Professional** | | |
| 18. How appropriate is it to provide patients after reaching the recovery threshold with access to a patient online forum, moderated by a qualified professional within the NHS TT service? | Face-to-face support helps overcome feelings of isolations which could contribute to symptoms of anxiety/depression. Whilst online groups ae beneficial, there are many that already exist. Having face-to face support provides support within the local community allowing you to build a network that can support you outside these sessions too. | In terms of the face-to-face support groups that my rating on, that I thought it was very appropriate came from my personal experiences where I felt the feeling of isolation really contributed to how poor I was feeling prior to being able to pursue care and feeling. (P10)  I think I would also be more in Favor of the in person group in general, and not the only because I feel like there is. There are already a lot of online forums, or or for or whatever how you call it like you can find, you can find online groups if you want, but in person groups, especially if they're like local groups that again, can signpost you to other local services or other. You know things that can be tangible in your specific community for me it would feel more relevant, more appropriate (PA5) |
| 20. How appropriate is it for patients after reaching the recovery threshold to access face-to-face support groups following end of treatment in talking therapy services? |  |  |
| **Section 5. Buddy support system – connecting patients of similar demographics backgrounds to help each other** | | |
| 19. How appropriate is it to connect two patients after reaching the recovery threshold with similar demographics and background to prevent relapse (i.e., a buddy support system)? | It has benefits but patients explored how this would be something that they would not take up dur to problems being more personal and having those conversations with another patient can be difficult. Also, his could cause comparison between recovery journeys which could be misleading. | I think I can see the pros and cons. And again, I think it would just be a very personal a very personal thing if that was something that an individual wanted to do or not, and it wouldn't be for everybody. (PA1)  But if it's a direct one on one body system, you almost. I would feel personally that my journey should mimic or should reflect their journey in some way. And that's very unlikely to happen. Everyone's journey to recovery. Everyone's journey through mental health looks different. And so I feel like. (P10) |
| **Section 7. Differences between lapse and relapse** | | |
| 26. How appropriate is it that patients know the difference between a lapse and a relapse after reaching the recovery threshold? | Discussion and clarification of the definition of lapse and relapse.  While its agreed that self-monitoring is extremely important due to the nature of the LiCBT treatment and the recovery journey which promotes independence, there needs to be resources and tools in place to help facilitate the self-monitoring.  Additionally, it was discussed that self-monitoring is important to help identify when a person is going through a lapse and relapse. One patent also mentioned how lapse vs relapse was not covered on their online CBT programme and the importance of having such an understanding of the distinction between terminology to aid their own recovery. | So I personally did not know the difference between lapse and relapse. But I am also not a native English speaker, so I think that plays a role in it as well. (Stella)  You are the only one who can actually measure how much you have recovered, and how much work that you still need to do (PA8)  if I'm to make real progress on getting to getting to a consistently healthier place, I need to realize when I'm entering back into those unhelpful ways of thinking (P10)  I guess from my experience of the sort of online programme was that … there was I guess, no real sort of discussion around that [terminology of lapse and relapse] or covering of that at all (PA1) |
| 27. How appropriate is it that patients’ regularly check in with themselves following treatment by recording/noting their mood? |  |  |
| **Section 7. Knowledge of current practice and processes** | | |
| 28. How appropriate is it that patients are knowledgeable about the current process when returning to service? | The patients expressed their concerns regarding the current referral process post-treatment, noting that it tends to be lengthy and complex. Consequently, the implementation of a more streamlined and accessible system would be advantageous. Additionally, some patients indicated a lack of clarity regarding the procedures involved in re-accessing services, emphasizing the need for improved communication and guidance in this area. | In regards to establishing an independent route to return to service, I think that’s quite valuable because when I first sought help for my difficulties, I went through my GP. I requested that my GP refer me to services which I then received a phone call back and it was something of a long and slightly convoluted process… I feel that anything that streamlines the ability to access help when I need it, that removes any potentially difficult stages (P10)  Personally, I don’t know that, I don’t know exactly still the process of returning to the service if and when I need it… so yeah, for me it would be extremely important to have clear signposting about initial access to the services. Return to the service. Like kind of streamline the whole process and make it easy to understand how you can access the service whether it's the 1st time, or if you're returning to it. (PA5)  So, perhaps a more straightforward route to accessing the services would be helpful for a lot of people. (PA5) |
| 29. How appropriate is it for talking therapy services to establish an independent route for patients reaching the recovery threshold, to return to service? |  |  |
