# Supplementary material for: Sustaining Recovery After Low‐Intensity Treatment for Anxiety and Depression in NHS Talking Therapies: A Multiphase Participatory and Consensus‐Building Study of Stakeholder Priorities and Recommendations
Source: Depress Anxiety. 2026 Jan 28;2026:9916526. doi: 10.1155/da/9916526 (PMC12852061; doi:10.1155/da/9916526)
Supplement: Supplementary file 5 — Supporting Information 5 File 3: Patient WS2 Discussion. This file presents a table that displays details of the moderated discussion of statements rated in disagreement following the first round of voting for Patient Workshop 2. The table includes the key discussion points and some illustrative quotes from participants. [file DA-2026-9916526-s003.docx]

**Supplementary File 3.**

*Patient workshop 2 overview of moderated discussion of statements with disagreement following round 1 voting and illustrative quotes.*

| **Statement** | **Key Discussion Points** | **Quotes** |
| --- | --- | --- |
| **How appropriate is it…** | | |
| **Section 1: Access to material after treatment** | | |
| 3) That patients receive or have access to materials/resources used during sessions after reaching the recovery threshold? | - Some, patients discussed how they continued to use the resources they used during treatment after treatment to help understand at what stage of their recovery they were at e.g., diary.  The resources are more effective when patients feel as though they are using it for their own benefit rather than to inform the therapist of their progress. Additionally, patients should understand the purpose of these resources to ensure they use them as needed  -Unless a pdf or physical copy is provided, online self-help platform such as Silvercloud have limited access which makes it difficult to use the same resources within treatment outside. | It’s appropriate because I’m still using the same system as what I was during the treatment now to sort of gauge where I am really and almost more to like to sort of identify and understand. If I'm going, you know, if I'm taking steps backwards. (PA12)  With the silvercloud as far as I remember, might have changed now, is that it's limited. So once you've finished, you only have so many weeks or months, and then your access ends. (PA13)  I didn't use any materials during my talking therapies. Well, I did. I had sheets to fill in what I'd done for various activities. But they're not anything that I've continued with. (PA11)  I just felt I was completing it just for the therapist. Well, I filled it in. But what was that all about? Because we haven't really discussed it?(PA11) |
| **Section 2: if anyone, who should be responsible for monitoring patients after reaching recovery?** | | |
| 8) That someone from NHS Talking Therapies services irrespective of whether they delivered treatment checks in with patients reaching the recovery threshold to monitor recovery? | -Check-ins are dependent on patient preferences.  -Whilst arranging these check-ins in advance so the patient is aware that they will be contacted following their treatment, it can have an opposite effect by increasing anxiety.  - A brief call without prior notice may be acceptable, but it is quite uncommon in an NHS setting, where formal notification is typically required to ensure that patients can prepare themselves. | I put neutral because it really depends on the patient and the level where they're being, because for example, for me sometimes I would be so anxious and stuff. If I was to be contacted again to check therapy while it will be great, it could probably do more harm than good, because it will just kind of send me down the rabbit hole. So that’s the only reason I'm like, I'm not sure exactly. I, you know, like, especially if I know in advance, I'll probably just get anxious. (P13)  I think if it was a phone call out of the blue or anything like that, I don't think I would have an issue with it. (P12) |
| **Section 3: Support from personal networks, GPs and local services** | | |
| 9) To involve social networks (friends, family, colleagues) in relapse prevention planning after reaching the recovery threshold? | Clarification regarding to what extent social networks will be involved.  The importance of considering confidentiality and ensuring nay involvement of external members is with patients’ consent. | I think maybe with regarding the social network, friends, families. I think they'd have to check in with the circumstances of the individual. (P12) |
| 10) To involve the GP or other healthcare professionals outside of NHS Talking Therapies services in relapse prevention planning after reaching the recovery threshold? | Important for the GP to be aware of the treatment that the patient has received. | I think that they [GP] should know… just for their records. They should probably know if I've been discharged in recovery. They should probably be aware of that. (P12) |
| 12) That NHS Talking Therapies services collaborate with local services in the health sector including GPs to provide care to patients after reaching the recovery threshold? | Clarification surrounding what falls under the category of local services e.g., community services, charities or other third sector organisations.  It is important to seek patients’ permission before approaching any service outside of Talking Therapies. |  |
| **Section 3: Support from local services and responsibility for initial contact** | | |
| 11) that NHS Talking Therapies services provide the INITIAL contact with external services that they signpost patients after researching recovery to address other/further needs? | It is important to ensure patient consent to being contacted and understand the external services they are being referred to which makes the initial process easier and quicker. This can be further detailed in the discharge letter.  If talking therapy services lack the capacity to share specific details—given that some external services prefer direct communication from patients—the therapist can assist the patient in navigating the referral process which can increase their confidence. | Perhaps with the agreement of the patient the details could be passed on to these outside services, and they could then send an invitation to take to join them to take part. I mean found it very difficult, I to make the 1st step to completing an application to take a course with the Wellbeing and Recovery College in my area. (P11)  I think it's also important that when the permission is asked of the patient that they understand, the patient understands why it would be beneficial. Not just like, Oh, yeah, you should do this, here's the link. (P13) |
| **Section 4: Incorporating additional roles within TT services** | | |
| 13) To include patient representatives within NHS Talking Therapies services to emphasise the importance of relapse prevention? | Clarification of what patient representatives mean. This could entail patients who drive the voice in for relapse prevention and advocate for relapse prevention within their services. It could include lived experience advisory boards. | I think it is a little bit confusing, patient representative is who, what is that exactly? (P12) |
| **Section 5: Support from experts by experience** |  |  |
| 19) For NHS Talking Therapies to connect two patients after reaching the recovery threshold with similar demographics and background to prevent relapse (i.e., a buddy support system) | Whilst it can be beneficial, it should not be excusive to those of the same background.  Prior to pairing two patients an introduction can be facilitated by a therapist to ensure both patients are comfortable socialising with each other independently.  Alternatively, a group setting might be more comfortable for some patients. | I think it’s very appropriate depending on how it’s done. Like if you pair me up with someone else, for example, and I don't know anything about them other than we're within the same demographic or background, I don't think that might necessarily be relevant. I think it should be more than just the background and stuff. I think it's also the context and the way that people think. I might have more in common with someone from a different background than what the therapist might know or understand. (P13)  Yeah, maybe you'd have to sort of have like an introduction, possibly with therapist maybe over a Zoom meeting with this other person and see how the two get on first before you, buddy them up. (P12)  When it's on a 1-to-one thing that can be much more difficult… I would probably find it easier initially to do a group, because then you're not so out there when you're just meeting one person when you've got to make a conversation. If you're in a group, you don't have to make conversation all the time. (P11) |
| **Section 5: Mediated support by NHS taking Therapies Professionals** | | |
| 20) For NHS Talking Therapies patients after reaching the recovery threshold to access face-to-face support groups following end of treatment in talking therapy services. | The support groups are helpful, but often, patients need struggle with initiating the first step. Therefore, having someone facilitate that transition is highly advantageous, as the guidance encourages patients to attend and enhances their confidence in engaging with external support groups. | It's like the you wanting to do the group therapy, a Handyman club. It's something that I think is a good idea. It's just like me taking that step to actually do it. I think that's the hard part… Did we say something earlier, a couple of couple of questions ago regarding the NHS sort of contacting external groups. Possibly again, maybe say, you know this, this person who's interested in joining the group and then reaching out to them, and then maybe they make the 1st move towards the patient.  (P12)  I think m the arrangements that have been made for when I start this group in January [recovery college referred by private therapist] by are that the person one of the people running it will meet me at the door… So what she has said is I'll meet you at the door and I'll show you the room that we're in. You won't have to walk down the corridor trying to figure out where to go. I will take you, and I was really pleased because that's a big help, because I thought I might get to the door and then chicken out and decide, no, I can't do this. (P11) |
| **Section 7: Differences between lapse and relapse, and self-monitoring** | | |
| 26) That patients know the difference between a lapse and a relapse after reaching the recovery threshold? | Clarification of the definition of lapse and relapse.  It is important for patients to be aware of the distinction between the two, but it can be difficult to reflect on what they are experiencing (a lapse or relapse) if they are currently in a difficult state.  Implementing techniques such as journaling can help individuals distinguish the intensity of their experiences, aiding in differentiating between a lapse and a relapse and also allowing them to prevent relapse in the future. | if I started to go back into like, you know, unhelpful cycles, It's difficult to differentiate the difference, you know, when you're actually in that mindset. Because, you know, it's I mean, you know, you possibly aren’t thinking clear, anyway, and you're in a  you know, it’s hard to sort of understand what's going on with yourself in a in that situation. So maybe I was thinking that it's less relevant, the difference between a lapse and a relapse when you're in that situation. (P12)  Journaling might be helpful, or learning how to, or even just having some sort of a teaching on how to get yourself to be more aware of your own patterns. And then and then try to basically think, learn. Someone to teach you how to notice these things yourself, ultimately. So then, in years later, or something that you'd know how to discern yourself, to prevent yourself, or even just to manage to get yourself out of it. (P13) |
