# Supplementary material for: Sustaining Recovery After Low‐Intensity Treatment for Anxiety and Depression in NHS Talking Therapies: A Multiphase Participatory and Consensus‐Building Study of Stakeholder Priorities and Recommendations
Source: Depress Anxiety. 2026 Jan 28;2026:9916526. doi: 10.1155/da/9916526 (PMC12852061; doi:10.1155/da/9916526)
Supplement: Supplementary file 6 — Supporting Information 6 File 4: Prof Ratings. This file presents two tables. The first table details individual professional/key stakeholder ratings (out of 9) of the appropriateness of each statement across workshops 1 and 2. The second table details individual professional and key stakeholder ratings per workshop for round 3 (out of 9) on the necessity of statements deemed appropriate and in agreement during round 2. [file DA-2026-9916526-s002.docx]

**Supplementary File 4:** Ratings of statements across professionals/key stakeholder workshops for each round

**Round 1 and Round 2**

| **Statements** | **Round 1 (ratings from 1 to 9)** | | | | | | | | | | **Round 2 (ratings from 1 to 9)** | | | | | | | | | |
| --- | --- | --- | --- | --- | --- | --- | --- | --- | --- | --- | --- | --- | --- | --- | --- | --- | --- | --- | --- | --- |
|  | **Workshop 1 (n=5)** | | | | | **Workshop 2 (n=5)** | | | | | **Workshop 1 (n=5)** | | | | | **Workshop 2 (n=5)** | | | | |
| **How appropriate is it …** | **PR2** | **PR4** | **PR5** | **PR6** | **PR7** | **PR1** | **PR9** | **PR10** | **PR13** | **PR14** | **PR2** | **PR4** | **PR5** | **PR6** | **PR7** | **PR1** | **PR9** | **PR10** | **PR13** | **PR14** |
| **Section 1. *Service level engagement to relapse prevention*** | | | | | | | | | | | | | | | | | | | | |
| 1) To support recovery as part of routine care after the patient has reached recovery threshold? | 8 | 9 | 9 | 9 | 9 | 9 | 9 | 8 | 9 | 8 | 8 | 9 | 9 | 9 | 9 | 9 | 9 | 8 | 9 | 9 |
| 2) To provide patients with a consolidation/maintenance period after reaching the recovery threshold? | 8 | 9 | 9 | 9 | 9 | 9 | 9 | 7 | 9 | 8 | 9 | 9 | 9 | 9 | 9 | 9 | 9 | 8 | 8 | 9 |
| 3) That patients receive or have access to materials/resources used during sessions after reaching the recovery threshold? | 9 | 9 | 9 | 9 | 9 | 9 | 9 | 8 | 9 | 9 | 9 | 9 | 9 | 9 | 9 | 9 | 9 | 9 | 9 | 9 |
| 4) That patients have access to new materials/resources after reaching the recovery threshold which have not been used during sessions? | 8 | 5 | 7 | 9 | 5 | 9 | 7 | 5 | 8 | 8 | 8 | 7 | 7 | 9 | 7 | 8 | 8 | 7 | 7 | 8 |
| **Section 3. External support after reaching the recovery threshold** | | | | | | | | | | | | | | | | | | | | |
| 5) To monitor clinical recovery after reaching the recovery threshold (using routine outcome measures including: PHQ-9, GAD-7, WSAS)? | 9 | 9 | 5 | 9 | 9 | 7 | 9 | 5 | 9 | 8 | 9 | 8 | 7 | 9 | 8 | 7 | 8 | 7 | 5 | 8 |
| 6) To assess personal recovery after reaching the recovery threshold? | 9 | 9 | 9 | 9 | 9 | 9 | 9 | 5 | 9 | 9 | 9 | 8 | 9 | 9 | 8 | 9 | 9 | 9 | 7 | 9 |
| 7) That the same person who delivered treatment checks in with the patient after reaching the recovery threshold to monitor recovery? | 5 | 7 | 5 | 9 | 7 | 8 | 9 | 5 | 9 | 7 | 8 | 8 | 7 | 9 | 7 | 4 | 5 | 7 | 7 | 6 |
| 8) That someone from NHS TT services irrespective of whether they delivered treatment checks in with patients reaching the recovery threshold to monitor recovery? | 5 | 6 | 5 | 9 | 5 | 6 | 9 | 5 | 8 | 9 | 5 | 6 | 7 | 8 | 7 | 7 | 8 | 7 | 6 | 7 |
| **Section 3. External support after reaching the recovery threshold** | | | | | | | | | | | | | | | | | | | | |
| 9) To involve social networks (friends, family, colleagues) in relapse prevention planning after reaching the recovery threshold? | 5 | 6 | 9 | 8 | 7 | 9 | 9 | 3 | 8 | 9 | 6 | 7 | 9 | 9 | 7 | 9 | 9 | 8 | 9 | 9 |
| 10) To involve the GP or other healthcare professionals outside of NHS TT services in relapse prevention planning after reaching the recovery threshold? | 5 | 5 | 9 | 9 | 9 | 9 | 9 | 5 | 9 | 8 | 6 | 7 | 9 | 9 | 7 | 9 | 9 | 8 | 9 | 8 |
| 11) That the NHS TT services provide INITIAL contact with external services that they signpost patients after reaching the recovery threshold, to address other needs? | 5 | 5 | 5 | 9 | 7 | 7 | 7 | 3 | 9 | 7 | 6 | 7 | 7 | 9 | 7 | 6 | 8 | 7 | 5 | 7 |
| 12) That NHS TT services collaborate and communicate with local services in the health sector including GPS to provide care to patients after reaching the recovery threshold? | 5 | 8 | 5 | 9 | 8 | 7 | 9 | 5 | 9 | 8 | 6 | 7 | 7 | 9 | 7 | 8 | 9 | 8 | 9 | 9 |
| **Section 4. Additional roles within TT services** | | | | | | | | | | | | | | | | | | | | |
| 13) To include patient representatives within NHS TT Services to emphasise the importance of relapse prevention? | 8 | 7 | 9 | 9 | 7 | 8 | 9 | 9 | 9 | 9 | 9 | 8 | 9 | 9 | 8 | 8 | 9 | 7 | 9 | 9 |
| 14) To develop a specific role within NHS TT services for relapse prevention after patients reached the recovery threshold? | 9 | 7 | 9 | 9 | 5 | 7 | 9 | 7 | 9 | 9 | 9 | 8 | 9 | 9 | 6 | 8 | 8 | 6 | 8 | 7 |
| **Section 5. Recommendations to maintain progress/wellbeing after reaching the recovery threshold** | | | | | | | | | | | | | | | | | | | | |
| 15) To provide refresher/booster courses for patients after reaching the recovery threshold to recap on treatment content? | 7 | 8 | 5 | 9 | 7 | 8 | 9 | 5 | 9 | 9 | 8 | 8 | 7 | 9 | 5 | 8 | 8 | 7 | 7 | 8 |
| 16) To have specific information in the NHS TT website for patients reaching the recovery threshold including information regarding local resources/online resources, links to external support services, preventing relapse etc? | 8 | 9 | 6 | 9 | 9 | 9 | 9 | 8 | 9 | 9 | 8 | 8 | 7 | 9 | 7 | 9 | 9 | 9 | 9 | 9 |
| 17) That the NHS TT services provide a 24-hour helpline for patients to connect with for a quick consultation regarding how to handle a particular situation causing symptoms of their anxiety/depression to resurface? | 1 | 7 | 4 | 9 | 2 | 4 | 3 | 2 | 8 | 6 | 2 | 3 | 3 | 9 | 1 | 1 | 1 | 1 | 2 | 2 |
| 18) To provide patients after reaching the recovery threshold with access to a patient online forum, moderated by a qualified professional within the NHS TT service? | 4 | 7 | 5 | 9 | 5 | 6 | 5 | 1 | 9 | 7 | 5 | 7 | 7 | 9 | 2 | 1 | 2 | 1 | 3 | 2 |
| 19) To connect two patients after reaching the recovery threshold with similar demographics and background to prevent relapse (i.e., a buddy support system)? | 2 | 6 | 4 | 9 | 2 | 4 | 5 | 1 | 5 | 5 | 3 | 5 | 3 | 9 | 4 | 4 | 5 | 5 | 7 | 2 |
| 20) For patients after reaching the recovery threshold to access face-to-face support groups following end of treatment in NHS TT services? | 6 | 6 | 4 | 9 | 5 | 6 | 6 | 1 | 8 | 7 | 5 | 5 | 3 | 9 | 5 | 7 | 3 | 4 | 4 | 2 |
| **Section 6. Awareness of guidelines and recommendations for relapse prevention:** | | | | | | | | | | | | | | | | | | | | |
| 21) That university training for PWPs captures recent policies, guidelines and recommendations surrounding relapse prevention? | 9 | 9 | 9 | 9 | 9 | 9 | 9 | 6 | 9 | 9 | 9 | 9 | 9 | 9 | 8 | 9 | 9 | 8 | 9 | 9 |
| 22) For clinical academics delivering university training for PWPs to be aware of up-to-date relapse prevention resources? | 9 | 9 | 9 | 9 | 9 | 9 | 9 | 8 | 9 | 9 | 9 | 9 | 9 | 9 | 8 | 9 | 9 | 9 | 9 | 9 |
| 23) That NHS TT staff delivering and supporting step 2 treatment are knowledgeable about policies, guidelines and recommendations surrounding relapse prevention? | 9 | 9 | 9 | 9 | 9 | 9 | 9 | 8 | 9 | 9 | 9 | 9 | 9 | 9 | 8 | 9 | 9 | 9 | 9 | 9 |
| 24) For NHS TT staff delivering and supporting step 2 treatment to be aware of up-to-date relapse prevention resources? | 9 | 9 | 9 | 9 | 9 | 9 | 9 | 8 | 9 | 9 | 9 | 9 | 9 | 9 | 8 | 9 | 9 | 9 | 9 | 9 |
| 25) For GPs and other external healthcare providers to be familiar with guidelines, policies and recommendations regarding relapse prevention in NHS TT services? | 9 | 9 | 9 | 9 | 7 | 9 | 9 | 7 | 9 | 9 | 8 | 9 | 9 | 9 | 7 | 9 | 9 | 8 | 9 | 9 |
| **Section 7. Expanding on training and ongoing discussions on relapse prevention:** | | | | | | | | | | | | | | | | | | | | |
| 26) That university training for PWPs expands on relapse prevention? | 7 | 9 | 9 | 9 | 8 | 9 | 9 | 9 | 9 | 8 | 9 | 9 | 9 | 9 | 7 | 9 | 9 | 9 | 9 | 9 |
| 27) To provide Continued Professional Development opportunities to for Psychological Wellbeing Practitioners following qualification to develop further knowledge and skills on relapse prevention? | 8 | 9 | 9 | 9 | 9 | 9 | 9 | 9 | 9 | 9 | 9 | 9 | 9 | 9 | 7 | 9 | 9 | 9 | 9 | 9 |
| 28) That relapse prevention is discussed during clinical supervision? | 8 | 9 | 9 | 9 | 9 | 9 | 9 | 9 | 9 | 9 | 9 | 9 | 9 | 9 | 8 | 9 | 9 | 9 | 9 | 9 |
| **Section 8. Patient knowledge and engagement with relapse prevention.** | | | | | | | | | | | | | | | | | | | | |
| 29) That patients know the difference between a lapse and a relapse after reaching the recovery threshold? | 9 | 9 | 8 | 9 | 7 | 9 | 9 | 8 | 9 | 9 | 9 | 9 | 9 | 9 | 7 | 9 | 9 | 8 | 9 | 9 |
| 30) That patients’ regularly check in with themselves following treatment by recording/noting their mood? | 7 | 9 | 8 | 9 | 8 | 9 | 9 | 7 | 7 | 9 | 8 | 8 | 9 | 9 | 7 | 9 | 9 | 8 | 9 | 9 |
| 31) That patients are knowledgeable about the current process when returning to service? | 6 | 9 | 9 | 9 | 9 | 9 | 9 | 9 | 9 | 9 | 8 | 9 | 9 | 9 | 7 | 9 | 9 | 9 | 9 | 9 |
| 32) For NHS TT services to establish an independent route for patients reaching the recovery threshold, to return to service? | 8 | 9 | 5 | 9 | 7 | 7 | 9 | 8 | 9 | 8 | 8 | 8 | 3 | 8 | 5 | 5 | 9 | 8 | 9 | 7 |

Note.

**Round 3 Ratings**

|  |  | | | | | | | | | |
| --- | --- | --- | --- | --- | --- | --- | --- | --- | --- | --- |
| **Statements** | **Round 3 (ratings from 1 to 9)** | | | | | | | | | |
|  | **Workshop 1 (n=5)** | | | | | **Workshop 2 (n=5)** | | | | |
| **How necessary is it …** | **PR2** | **PR4** | **PR5** | **PR6** | **PR7** | **PR1** | **PR9** | **PR10** | **PR13** | **PR14** |
| **Section 1. *Service level engagement to relapse prevention*** |  |  |  |  |  |  |  |  |  |  |
| 1) To support recovery as part of routine care after the patient has reached recovery threshold? | 9 | 7 | 9 | 9 | 8 | 9 | 9 | 9 | 9 | 9 |
| 2) To provide patients with a consolidation/maintenance period after reaching the recovery threshold? | 9 | 8 | 8 | 9 | 8 | 7 | 8 | 8 | 8 | 8 |
| 3) That patients receive or have access to materials/resources used during sessions after reaching the recovery threshold? | 9 | 8 | 9 | 9 | 8 | 8 | 9 | 9 | 9 | 9 |
| 4) That patients have access to new materials/resources after reaching the recovery threshold which have not been used during sessions? | 5 | 5 | 7 | 9 | 7 | 6 | 8 | 7 | 7 | 7 |
| **Section 3. External support after reaching the recovery threshold** |  |  |  |  |  |  |  |  |  |  |
| 5) To monitor clinical recovery after reaching the recovery threshold (using routine outcome measures including: PHQ-9, GAD-7, WSAS)? | 9 | 6 | 7 | 7 | 8 | - | - | - | - | - |
| 6) To assess personal recovery after reaching the recovery threshold? | 9 | 8 | 7 | 9 | 8 | 9 | 9 | 9 | 8 | 9 |
| 7) that the same person who delivered treatment checks in with the patient after reaching the recovery threshold to monitor recovery? | 5 | 7 | 3 | 9 | 7 | - | - | - | - | - |
| 8) That someone from NHS TT services irrespective of whether they delivered treatment checks in with patients reaching the recovery threshold to monitor recovery? | - | - | - | - | - | - | - | - | - | - |
| **Section 3. External support after reaching the recovery threshold** |  |  |  |  |  |  |  |  |  |  |
| 9) To involve social networks (friends, family, colleagues) in relapse prevention planning after reaching the recovery threshold? | - | - | - | - | - | 9 | 9 | 8 | 9 | 8 |
| 10) To involve the GP or other healthcare professionals outside of NHS TT services in relapse prevention planning after reaching the recovery threshold? | - | - | - | - | - | 6 | 9 | 8 | 8 | 7 |
| 11) That the NHS TT services provide INITIAL contact with external services that they signpost patients after reaching the recovery threshold, to address other needs? | - | - | - | - | - | - | - | - | - | - |
| 12) That NHS TT services collaborate and communicate with local services in the health sector including GPS to provide care to patients after reaching the recovery threshold? | - | - | - | - | - | 7 | 9 | 7 | 9 | 8 |
| **Section 4. Additional roles within TT services** |  |  |  |  |  |  |  |  |  |  |
| 13) To include patient representatives within NHS TT Services to emphasise the importance of relapse prevention? | 7 | 6 | 9 | 7 | 7 | 7 | 9 | 7 | 7 | 9 |
| 14) To develop a specific role within NHS TT services for relapse prevention after patients reached the recovery threshold? | - | - | - | - | - | - | - | - | - | - |
| **Section 5. Recommendations to maintain progress/wellbeing after reaching the recovery threshold** |  |  |  |  |  |  |  |  |  |  |
| 15) To provide refresher/booster courses for patients after reaching the recovery threshold to recap on treatment content? | - | - | - | - | - | 7 | 8 | 7 | 7 | 9 |
| 16) To have specific information in the NHS TT website for patients reaching the recovery threshold including information regarding local resources/online resources, links to external support services, preventing relapse etc? | 7 | 8 | 9 | 3 | 7 | 9 | 9 | 9 | 9 | 9 |
| 17) That the NHS TT services provide a 24-hour helpline for patients to connect with for a quick consultation regarding how to handle a particular situation causing symptoms of their anxiety/depression to resurface? | - | - | - | - | - | - | - | - | - | - |
| 18) to provide patients after reaching the recovery threshold with access to a patient online forum, moderated by a qualified professional within the NHS TT service? | - | - | - | - | - | - | - | - | - | - |
| 19) To connect two patients after reaching the recovery threshold with similar demographics and background to prevent relapse (i.e., a buddy support system)? | - | - | - | - | - | - | - | - | - | - |
| 20) For patients after reaching the recovery threshold to access face-to-face support groups following end of treatment in NHS TT services? | - | - | - | - | - | - | - | - | - | - |
| **Section 6. Awareness of guidelines and recommendations for relapse prevention:** |  |  |  |  |  |  |  |  |  |  |
| 21) That university training for PWPs captures recent policies, guidelines and recommendations surrounding relapse prevention? | 9 | 8 | 9 | 9 | 8 | 9 | 9 | 9 | 9 | 9 |
| 22) For clinical academics delivering university training for PWPs to be aware of up-to-date relapse prevention resources? | 9 | 8 | 9 | 9 | 7 | 9 | 9 | 9 | 9 | 9 |
| 23) That NHS TT staff delivering and supporting step 2 treatment are knowledgeable about policies, guidelines and recommendations surrounding relapse prevention? | 9 | 8 | 9 | 9 | 7 | 9 | 9 | 9 | 9 | 9 |
| 24) For NHS TT staff delivering and supporting step 2 treatment to be aware of up-to-date relapse prevention resources? | 9 | 8 | 9 | 9 | 7 | 9 | 9 | 8 | 9 | 9 |
| 25) For GPs and other external healthcare providers to be familiar with guidelines, policies and recommendations regarding relapse prevention in NHS TT services? | 7 | 7 | 9 | 9 | 3 | 9 | 9 | 7 | 9 | 8 |
| **Section 7. Expanding on training and ongoing discussions on relapse prevention:** |  |  |  |  |  |  |  |  |  |  |
| 26) That university training for PWPs expands on relapse prevention? | 9 | 8 | 9 | 9 | 3 | 8 | 9 | 9 | 9 | 9 |
| 27) To provide Continued Professional Development opportunities to for Psychological Wellbeing Practitioners following qualification to develop further knowledge and skills on relapse prevention? | 7 | 7 | 9 | 9 | 7 | 5 | 9 | 8 | 9 | 8 |
| 28) That relapse prevention is discussed during clinical supervision? | 8 | 7 | 9 | 9 | 7 | 9 | 9 | 8 | 9 | 9 |
| **Section 8. Patient knowledge and engagement with relapse prevention.** |  |  |  |  |  |  |  |  |  |  |
| 29) That patients know the difference between a lapse and a relapse after reaching the recovery threshold? | 9 | 8 | 8 | 9 | 3 | 7 | 9 | 8 | 9 | 9 |
| 30) That patients’ regularly check in with themselves following treatment by recording/noting their mood? | 6 | 8 | 8 | 9 | 7 | 8 | 9 | 7 | 8 | 9 |
| 31) That patients are knowledgeable about the current process when returning to service? | 7 | 7 | 8 | 9 | 3 | 6 | 8 | 9 | 9 | 9 |
| 32) For NHS TT services to establish an independent route for patients reaching the recovery threshold, to return to service? | - | - | - | - | - | - | - | - | - | - |

Note. - = Note Rated.
