# Supplementary material for: Sustaining Recovery After Low‐Intensity Treatment for Anxiety and Depression in NHS Talking Therapies: A Multiphase Participatory and Consensus‐Building Study of Stakeholder Priorities and Recommendations
Source: Depress Anxiety. 2026 Jan 28;2026:9916526. doi: 10.1155/da/9916526 (PMC12852061; doi:10.1155/da/9916526)
Supplement: Supplementary file 7 — Supporting Information 7 File 5: Prof WS1 Discussion. This file presents a table that displays details of the moderated discussion of statements rated in disagreement following the first round of voting for the professional and key stakeholders’ workshop 1. The table includes the key discussion points and some illustrative quotes from participants. [file DA-2026-9916526-s001.docx]

**Supplementary File 5.**

*Professionals/Key stakeholders Workshop 1 overview of the moderated discussion of statements with disagreement following round 1 voting and illustrative quotes.*

| **Statement** | **Key discussion points** | **Quotes** |
| --- | --- | --- |
| **How appropriate is it …** |  |  |
| 4) That patients have access to new materials/resources after reaching the recovery threshold which have not been used during sessions? | - Resources vary across patients and tailored to their needs, so not all resources will be helpful for all patients.  - It is integral that patients understand the material provided before using it, and if this is provided after the session, then they do not have access to their therapist to explain it.  - Confusion around language surrounding appropriateness and access.  -  Providing patients with various materials is appropriate to ensure they can access anything not discussed within the short sessions. | *I'm not sure what the disagreement, if there is a disagreement, or whether it is around language, you know, this appropriateness and access* (PR2)  *It’s variable in the patient… But it's very much about, you know, your patient. So, would that work? What might work for one might not for another. And that's why I went down the middle.* (PR4)  *I think it’s more around having like the availability and range so like it can be helpful, but some of the materials and resources might cover other things that, especially at step-2 level, we weren’t able to really kind of like work on. … I think it's appropriate for there to be like access* (PR5) |
| 5) To monitor clinical recovery after reaching the recovery threshold (using routine outcome measures including: PHQ-9, GAD-7, WSAS)? | - It is appropriate to monitor recovery after treatment, but this does not have to be done with routine outcome measures.  - approach clinical recovery following treatment using a more qualitative approach.  - The patient could monitor their recovery by checking in with themselves using yes/no questions, e.g., "Am I still doing this, or have I stopped XYZ?" | I picked extremely appropriate because it goes back to the fact that those questionnaire scores aren’t everything. So it’s, I still feel for long term recovery. It’s helpful to check in even if someone seems to be OK and well, you don’t know what’s going to happen (PR4)  I guess for me it’s kind of looking at the language of the of the question. So, it’s like how appropriate is it to monitor clinical recovery after reaching the recovery threshold using those measures like I don’t think it always has to be done like with the measures … It could be a conversation about like, what do you like, and what do you notice? (PR5) |
| 7) that the same person who delivered treatment checks in with the patient after reaching the recovery threshold to monitor recovery? | - Importance of the therapeutic relationship  - Highly dependent on the patients’ preference and the service capacity | *I went neutral, probably clearly for the simple thing that I actually feel quite like neutral about it… So I feel like although it could be a good thing, like putting a kind of precedence on like an importance on it being the same person and then the patient being told that it would be the same person feels a bit like, It could end being a bit disastrous.* (PR5)  *Perhaps it depends on what you think the active ingredient of all this is. So, if and it might be different for different people, but if the active ingredient is relationship and that's what actually has helped them to get better and it's not very much to do with the other stuff… then maybe and I know it's inconvenient for the service and I don't know how you get around that, but maybe it should be with the same person. But I put neutral because I thought well, let the patients choose if that's a possibility.* (PR2) |
| 8) That someone from NHS Talking Therapies services irrespective of whether they delivered treatment checks in with patients reaching the recovery threshold to monitor recovery? | - Follow-up with the patient is crucial, and it is best if the same professional who delivered the treatment does it. This approach ensures continuity in care and avoids the follow-up seeming like a routine task.  - However, having a different person could offer an opportunity to explore other things.  - The practicalities of having someone different, as not all professionals will continue to work in the service. | *Perhaps does depend on what you're trying to get, because I think there is a risk. It could be a tick box exercise if questions are asked in a certain way. But it would also give an opportunity to ask a few different questions, and the person might feel able to be more honest.* (PR2)  I went extremely appropriate because I do feel that a call, a follow-up call to monitor recovery is again a great idea. (PR6)  …and obviously practicalities therapists move on. Personal well-being. Practitioners move on. (PR7) |
| 9) To involve social networks (friends, family, colleagues) in relapse prevention planning after reaching the recovery threshold? | - Social network involvement could be passive or active, whereby they know they are part of the support plan or their presence alone provides support, but they do not know that.  - dependent on patient preferences and whether they discuss mental health with others.  - Family and friends can help identify change and are part of the patients’ lives longer than their therapists. | - *I find when I work with people, it's quite a divide. Some people really do involve friends and family in their mental health and others really don't want to* (PR4)  *it’s like the social network and the people around them are the people who are going to be around like longer than I am, I guess… I guess it’s kind of like the importance of the relationships and the networks that they have and that connection… It's usually family members or partners who have noticed the changes, who have noticed there's something wrong and they're like, oh, well such and such. So in fact, actually having an extra person to be able to notice that you maybe stop doing things feels like it can be really important, because sometimes a lot of the people like, a lot of times people don't really notice until, like they're being told or like, they're like they've slipped back down into a relapse.* (PR5) |
| 10) To involve the GP or other healthcare professionals outside of Talking Therapies services in relapse prevention planning after reaching the recovery threshold? | - Dependent on patient preferences  - The involvement of other healthcare professionals may be more beneficial than the GP ensuring the patient is aware that they are part of their relapse prevention plan. | *I was thinking not in terms of necessarily GP, but where if you’ve got someone who’s open to kind of like drug and alcohol services like midwives, health visitors, people who are gonna kind of like continually kind of, and it could just be kind of like a conversation and making them aware that … they're going to be part of the relapse prevention plan, so it doesn't become kind of like a shock to them.* (PR5)  *- I think it just really, it's, its patient led. So, if they said they wanted that, then you would support them with that. But sometimes we get patients that really don't want that*. (PR4) |
| 11) that the Talking Therapies services provide INITIAL contact with external services that they signpost patients after reaching the recovery threshold, to address other needs? | - Although professionals deemed this appropriate, the disagreement lay in the fact that not all patients would want that. | *it is like an appropriate kind of like I feel like it is an appropriate thing to do, but it doesn't necessarily have to be done for kind of like everyone does it?* (PR5)  *I think it is extremely appropriate because obviously you hand out these numbers to patients following treatment, it doesn't necessarily mean that they're going to make contact. They're not going to make use of the numbers.* (PR6) |
| 12) That Talking Therapies services collaborate and communicate with local services in the health sector including GPS to provide care to patients after reaching the recovery threshold? | - Enhanced communication between services increases knowledge of what the services offer.  -Although there were some reservations, it would have been appropriate if there had been more personalisation and planning. | *if you have good communication with other services, you just know a bit more about it, don't you? And then you can feel a bit more passionate about referring someone on to it and having those discussions with the person that you're working with*. (PR7) |
| 14) To develop a specific role within NHS TT services for relapse prevention after patients reached the recovery threshold? | - Revelation that a relapse prevention team is present in one service, and discussions revolve around what the team does | *I don't have actual statistics, but I think that it is really it is working really well because we've had it for a while now* (PR6) |
| 16) To have specific information in the NHS TT website for patients reaching the recovery threshold including information regarding local resources/online resources, links to external support services, preventing relapse etc? | -Mistakenly rated inappropriate, but having all information somewhere, such as a website, was deemed appropriate. | *I'm not really sure why I put the five. But would be happy to kind of like to move it up.* (PR5) |
| 17) That the NHS TT services provide a 24-hour helpline for patients to connect with for a quick consultation regarding how to handle a particular situation causing symptoms of their anxiety/depression to resurface? | - the importance of clarifying to patients the difference between what the helpline offers and how it differs from a crisis helpline.  - considerations for the management of risk and how it could become unmanageable.  - A helpline would go against the remit of talking therapies, in which the patient learns to manage their symptoms independently, and could be used as a form of reassurance-seeking by patients. | I feel like if the if the helpline was if patients were made aware that it wasn't a crisis helpline, it's literally just a conversation, consultation, etcetera, that it could be really effective. (PR6)  *It kind of almost takes away what like talking therapies remit kind of is and the work that we kind do, and it feels to me like it could almost like promote quite a lot of kind of like reassurance seeking*. (PR5)  *I scored it a one for the for that the risk sort of. And how on Earth you would manage it really. Maybe not a helpline. Maybe a little tiny video for kind of for each different problem descriptor or presentation that they could kind of just access and look up.* (PR2) |
| 18) to provide patients after reaching the recovery threshold with access to a patient online forum, moderated by a qualified professional within the NHS TT service? | -The statement is vague, which is why the neutral rating lies.  - lack of information regarding how this would be operationalised and implemented. | I do think it would be really appropriate. I think obviously, it's talking therapy, isn't it? And if people have the choice to join a forum and speak about their problems, I do see the benefits in it. (PR6)  *My thoughts are around, I guess the purpose of the online forum because with the professional way of seeing it, I suppose that kind of might lend itself to certain expectations or intentions, or you know whether it's around people being able to connect with each other and a bit more of social support.* (PR5) |
| ) To connect two patients after reaching the recovery threshold with similar demographics and background to prevent relapse (i.e., a buddy support system)? | -It is important to find a connection, but this could be done independently or outside of the service.  - dependent on patient preferences | *I think it could be a really helpful idea for certain people and again, it's always about that opinion, that some people might like it, and others might not.* (PR4)  *I've often thought you know that this sort of thing would be a really good idea, I think probably just become a bit risk averse, you know, and kind of concerned that you connect two people and it seems like a great idea, but actually you know, finding people that you connect with in a helpful way isn't always that predictable. So I kind of veered away from thinking it was a good idea.* (PR2) |
| 20) For patients after reaching the recovery threshold to access face-to-face support groups following end of treatment in talking therapy services? | - Logistical and geographical issues  - Dependent on patient preferences and offering multiple formats that are not exclusively face-to-face. | *probably kind of like logistics, like where we are … how Cumbria is kind of set out. I feel that if it were going to be offering some kind of groups, they would not like, they would end up not being face to face… Just to kind of like geography of the area. (*PR5)  *Some people will benefit more from it being face to face, and I understand that, but I don't think it has to be or yeah.* (PR4) |
| 31) That patients are knowledgeable about the current process when returning to service? | - misunderstanding regarding wording as the term 'appropriate' was switched with 'essential'  - clarifying expectations beforehand and ensuring patients know how the stepped-care procedures work. | *I probably didn't give it quite enough thought actually. And I think maybe I misunderstood or felt the word was instead of appropriate essential. You know, actually I think it is appropriate. But it's perhaps not something everybody can know or will know, but I think it probably is highly appropriate. So I probably didn't really think of it quite clearly enough.* (PR7)  *I scored 9 because when a patient comes back into the service, they expect to be placed on the step three wait list straight away and we have to explain that you know, you must follow the step care procedure and the model and you know sometimes isn’t made clear to the patients which means they’ve got these false expectation for what they want.* (PR6) |
| 32) For talking therapy services to establish an independent route for patients reaching the recovery threshold, to return to service? | -Misunderstanding of the question leading to a neutral response. | Think I might have read this question wrong, in terms of when it was an independent route, I kind of thought self-referral. (PR7) |
