# Supplementary material for: Sustaining Recovery After Low‐Intensity Treatment for Anxiety and Depression in NHS Talking Therapies: A Multiphase Participatory and Consensus‐Building Study of Stakeholder Priorities and Recommendations
Source: Depress Anxiety. 2026 Jan 28;2026:9916526. doi: 10.1155/da/9916526 (PMC12852061; doi:10.1155/da/9916526)
Supplement: Supplementary file 8 — Supporting Information 8 File 6: Prof WS2 Discussion. This file presents a table that displays details of the moderated discussion of statements rated in disagreement following the first round of voting for the professional and key stakeholders’ workshop 2. The table includes the key discussion points and some illustrative quotes from participants. [file DA-2026-9916526-s007.docx]

**Supplementary File 6.**

*Professionals/Key Stakeholders Workshop 2 Overview of the moderated discussion of statements with disagreement following round 1 voting and illustrative quotes.*

| **Statement** | **Key discussion points** | **Quotes** |
| --- | --- | --- |
| **How appropriate is it …** |  |  |
| **SECTION 1: Accessing new material after treatment (i.e., not used during sessions)** | | |
| 4) That patients have access to new materials/resources after reaching the recovery threshold which have not been used during sessions? | Discussions highlighted how new resources to patients presents a distinct challenge, particularly when these resources have not been previously established within their care context. This lack of familiarity can hinder patients' ability to independently comprehend and utilise these resources effectively.  Conversely, the presentation of new resources holds significant potential to encourage patients to engage with the concept of long-term recovery. By highlighting these options, healthcare providers can facilitate a shift in focus toward the maintenance of progress made during treatment. | if you're giving them new things, how easy are they going to find it to understand and how accessible, is it going to be and should everything at least have been introduced, rather than it being new? (PR14)  I felt like bringing some new materials might be quite supportive just in terms of that kind of shift of perspective and actually thinking about longevity. (PR1) |
| **SECTION 2: monitoring and assessing recovery after reaching recovery threshold** | | |
| 5) to monitor clinical recovery after reaching the recovery threshold (using routine outcome measures including: PHQ-9, GAD-7, WSAS)? | Discussions highlighted the lack of confidence among practitioners in continuing to use the routine outcome measures after treatment as opposed to other measures, which are less symptom-focused and more concerned about well-being, e.g., WEMWBS  Discussions emphasised the importance of the RAND/UCLA methodology, which does not consider feasibility at the first stage, just the appropriateness of each suggestion. | Particularly if we think about risk and carrying, if we're checking in with patients, are they then still technically on a caseload. So in terms of managing risk, managing safety, having a responsibility, clinical responsibility, I can see how there would be resistance to this and would we monitor that over time, whose responsibility is it? (PR9) |
| 6) to assess personal recovery after reaching the recovery threshold? |  |  |
| **Section 2: if anyone, who should be responsible for monitoring patients after reaching recovery?** | | |
| 7) that the same person who delivered treatment checks in with the patient after reaching the recovery threshold to monitor recovery? | - From the patient's perspective, monitoring should be conducted by someone who has established a therapeutic relationship with them, as this demonstrates a genuine commitment to their care and recovery  However, as there is high movement and progression of PWPs within talking therapies, it may make it challenging to ensure the same practitioner follow-up with the patient.  Although it would be a patient-centred approach, it would be challenging to coordinate this within current staff schedules.  Discussions raised concerns about becoming overly dependent on specific practitioners, which could overshadow the importance of empowering patients and the effectiveness of various treatment techniques.  Having a different practitioner could be beneficial for those who struggled with their therapeutic alliance during treatment, which impacted engagement and contributed to relapse, as it would provide an opportunity to disclose their problems and barriers in a conformable manner. | I was a high scorer on this because thinking about it from the patient's perspective, I think if somebody's going to check in on you, you want it to be the person that you formed a therapeutic relationship with rather than it just be a service thing because it could feel a little bit tick boxy if it was somebody else. (PR9)  ideally I think that would be lovely, but I can't see how in real life it would be happening... And I think ideally it would be the same practitioner if that were possible. (PR10)  I do actually think there may be potential benefits to working with a different practitioner … but sometimes I would get comments on patients like oh, of course, you know I can’t imagine working with anyone but you. And I think sometimes that does maybe give a sign that actually the mechanism of change in treatment is maybe less about the techniques and less abut empowering the patient to make those changes and maybe a bit more on that dependency on the practitioner, which I think can be a bit of a concern. (PR1) |
| 8) that someone from NHS TT services irrespective of whether they delivered treatment checks in with patients reaching the recovery threshold to monitor recovery? |  |  |
| **SECTION 3: Section 3: Support from personal networks, GPs and local services** | | |
| 9) To involve social networks (friends, family, colleagues) in relapse prevention planning after reaching the recovery threshold? | - Discussions highlighted how the inclusion of social support ensures close adherence to NICE guidelines.  -Ongoing involvement in social networks throughout and beyond treatment encourages patients to utilise available resources, increasing their confidence and comfort in engaging with those around them.  -Clarification surrounding a misunderstanding that the involvement of social networks is through the service after the treatment rather than being a part of the treatment discussions. | in my mind I was just thinking about NICE guidelines, particularly in the context of the recent changes in terms of risk assessment, risk management, risk review and obviously there's a much greater involvement of support networks (PR9)  actually it is really important for people to look at what support networks they have around them and that might not be the traditional sense (PR9)  if it's sort of a part of that sort of planning phase when. Therapies ended then obviously, yeah, I think all of those things are are very appropriate. (PR10) |
| 10) To involve the GP or other healthcare professionals outside of NHS TT services in relapse prevention planning after reaching the recovery threshold? | -GPs are seen as the initial point of contact for patients and their involvement ensures cohesive support and facilitates timely interventions for patients. | I feel by including GPs into relevant relapse prevention frameworks, I just think it builds that more cohesive network, so it allows us to provide the help for the people who need it the most and do as efficiently an as quickly as possible. (PR13) |
|  |  |  |
| 12) That NHS TT services collaborate and communicate with local services in the health sector including GPS to provide care to patients after reaching the recovery threshold? | There is a disconnect between talking therapies and local community services, which often do not address specific needs related to relapse prevention. Bridging this gap is essential for fostering a holistic approach to mental health care that effectively responds to patients' diverse needs and promotes sustained recovery. | there is that kind of, I guess disconnect between the talking therapies, but then also what's offered on a local social community level. So, it's just to bridge that gap to making sure it really aligns with relapse prevention that is talked about in the talking therapy as well, if it is even talked about to an extensive extent because the issue local services don't necessarily maybe address some of the specifics that people may need. (PR13) |
| **SECTION 3: Support from local services and responsibility for initial contact.** | | |
| 11) that the NHS TT services provide INITIAL contact with external services that they signpost patients after reaching the recovery threshold, to address other needs? | -Discussions highlighted how support from local services and the responsibility for initial contact are essential for providing continuity of care. This approach prevents patients from having to repeat their medical histories to multiple practitioners throughout their recovery journey.  - Practitioners suggested that this could be implemented on a needs basis rather than being required for everyone  - Some external services may prefer that the patient refer themselves to gain a first-hand understanding of their experience, indicating their engagement and motivation moving forward.  - In events where direct referrals are not possible, one practitioner highlighted how they sit with patients and go through exactly how to make that referral themselves, which has been beneficial. This can also help vulnerable patients complete online forms to disclose everything in a manner that effectively summarises their experiences with someone whom they have built trust with, guiding and encouraging them through the process.  - Discussions also highlighted how it is important to encourage autonomy to help patients build confidence and succeed in their recovery journey. | I think it can be good practice because I know something that a lot of patients report is often that they feel like they're just going around the houses of services… so I think in terms that continuity of care, I think it can be really useful for the patient experience to, you know, have someone, I guess, make that initial contact or just have that kind of linking up. And I think particularly if you've been working with someone for a number of sessions well, you might have insights into, you know, their engagement and the things that have been useful and supportive that you may be able to pass on to that service as well to set them up. (PR1)  So I completely agree that it would support the patient, particularly if they have been passed around a lot. But I just wonder about the practicalities of it and whether we are then taking away some form of independence if we are doing that for everybody. So I was thinking it might be on a needs basis is sort of where my mind was with that one. (PR9)  Also, some of our local services don't accept a referral from our service. The patient would have to self-refer. (PR10)  I think a key focus should be trying to encourage that autonomy within patients 'cause I feel like that would also in terms of encouraging that autonomy, that will make them feel better about themselves and realize, oh, I can do it. Like it's not someone who's, like holding my hand doing it, like I can actually do it. So, it shows that recovery is truly taking place. (PR13) |
| **Section 5: Support by NHS Talking Therapies following Treatment** | | |
| 15) to provide refresher/booster courses for patients after reaching the recovery threshold to recap on treatment content? | - Clarification of statement on whether this option and recommendation is meant to be offered to everyone.  - Discussions about the resource implications that should be considered e.g., who will deliver the service and how it will be delivered. This was important as professionals mentioned avoiding creating a dependency or giving the impression that ongoing visits to a trained practitioner are necessary.  - One practitioner from previous experience highlighted that certain services provide a one-time group session between one and three months after discharge to revisit techniques. This approach was beneficial for reinforcing concepts that may have been forgotten in a group setting. | I guess some of it does depend on things like timing, and I guess there's also a resource implication as to how you deliver it and who delivers it and really thinking about what the content of that needs to look like (PR14)  Giving people a bit of a booster because as much as they've learnt the skills, obviously we all know that if you don't practice something continually that you sometimes lose the skill and they just forget. (PR9) |
| 17) That the NHS TT services provide a 24-hour helpline for patients to connect with for a quick consultation regarding how to handle a particular situation causing symptoms of their anxiety/depression to resurface? | Discussions focussed on how provision of continuous 24-hour access to certain resources may be deemed inappropriate, as such access does not necessarily align with the actual needs of individuals. Also, this can result in misunderstandings regarding the offerings and intended purpose of the 24-hour helpline.  Such misinterpretations regarding the purpose of the helpline may contribute to an increase in reassurance-seeking behaviours among patients, potentially undermining the effectiveness of the service. | I just what it will be offering is not something that you need 24-hour access to and I just think it would muddy the water about what a 24 hour helpline was about in a way that would also be really unhelpful to other services that do need to be 24 hours (PR14)  I just don't think that that would be helpful. I think it could, it almost undermines all the work that you're doing with the patient to try and enable them to manage their own anxiety (PR9) |
| **Section 5: Mediated support by NHS Talking Therapies Professionals** | | |
| 18) to provide patients after reaching the recovery threshold with access to a patient online forum, moderated by a qualified professional within the NHS TT service? | -The provision of support groups was discussed as being outside the remit of NHS Talking Therapies which do not provide life-long support but rather short-term help. This inclusion within the existing talking therapies framework may add complications regarding how it will function in current service provision.  -Ther are also other support groups available that are more adept at facilitating that informal support and catering to the diverse needs of patients.  Support groups could present a valuable opportunity for participants to broaden their social networks and enhance their interpersonal connections. | *But I'm not sure that the talking therapy service will ever have the results or capacity to manage kind of ongoing informal support groups (PR14)*  I think the way I thought about the face to face support groups is because obviously it's a group, so I just thought that's a method to expand the social network, which is obviously expanding the social support of individuals.(PR13) |
| 20) For patients after reaching the recovery threshold to access face-to-face support groups following end of treatment in talking therapy services? |  |  |
|  |  |  |
| **SECTION 6: Awareness of policies and guidelines** |  |  |
| 21) that university training for Psychological Wellbeing Practitioners captures recent policies, guidelines and recommendations surrounding relapse prevention? | - A recent observation by a clinical academic delivering training underscored the noticeable lack of understanding of policies and legislation by current trainees, particularly within essays despite the constant mention of such key documents throughout training.  - It is essential for trainees to develop a comprehensive understanding of the legal implications and the professional and ethical guidelines that underpin their competencies. | I think that in my mind, I think I sort of understood it, but I was kind of thinking well in terms of policies and guidelines, I guess I was thinking on a broader level, perhaps because of my job remit, but around like local policies in terms of NHS. (PR10)  what we're seeing in essays in sort of generally anything that involves the our trainees bring in their theoretical underpinning is that they're missing a lot of this. We're constantly talking about it right from induction we bring it all the way through teaching. We're referencing different policies and legislation, and we're just not seeing trainees engaging with it and fully understanding sometimes why they have certain skills, for instance, why they have a shared understanding why COM-B is so important, even though we've taught it all the way through the course. (PR9) |
